# A Ridge-Regularized Jackknifed Anderson-Rubin Test

**DOI:** 10.1080/07350015.2023.2290739

**Published:** 2023-12-12

**Authors:** Max-Sebastian Dovì, Anders Bredahl Kock, Sophocles Mavroeidis

**Affiliations:** a International Monetary Fund, Washington, DC; b Department of Economics, University of Oxford, Oxford, UK

**Keywords:** High dimensional models, Instrumental variables, Ridge regression, Weak identification

## Abstract

We consider hypothesis testing in instrumental variable regression models with few included exogenous covariates but many instruments—possibly more than the number of observations. We show that a ridge-regularized version of the jackknifed Anderson and Rubin (henceforth AR) test controls asymptotic size in the presence of heteroscedasticity, and when the instruments may be arbitrarily weak. Asymptotic size control is established under weaker assumptions than those imposed for recently proposed jackknifed AR tests in the literature. Furthermore, ridge-regularization extends the scope of jackknifed AR tests to situations in which there are more instruments than observations. Monte Carlo simulations indicate that our method has favorable finite-sample size and power properties compared to recently proposed alternative approaches in the literature. An empirical application on the elasticity of substitution between immigrants and natives in the United States illustrates the usefulness of the proposed method for practitioners.

## 1 Introduction

Instrumental variables (IVs) are commonly employed in economics and related fields to estimate causal effects from observational data. The use of a large number of IVs has gained popularity due to weak identification, where researchers aim to capture limited exogenous variation in endogenous covariates and obtain more precise inference. One example is Mendelian randomization in biology, where weakly associated genetic mutations are used as IVs, and the number of IVs can exceed the number of observations (Davies et al. 2015; Burgess and Thompson 2021). Other prominent examples are the granular IV approach (Gabaix and Koijen 2020) and the saturation approach to identify treatment effects (Blandhol et al. 2022).^1^ However, it is crucial to have reliable inference methods that account for the potential lack of joint informativeness of a large number of weak IVs, especially when pretesting the strength of IVs is impractical or not available due to the excessive number of IVs compared to observations. This article contributes to the literature on the development of such methods.

We propose a ridge-regularized jackknifed Anderson-Rubin (RJAR) test to construct confidence sets for the coefficients of endogenous variables in weakly-identified and heteroscedastic IV models when the number of IVs is large. Jackknife-based methods have recently been used in this context because they are applicable in an asymptotic framework where the number of IVs diverges with the number of observations. However, by relying on existing central limit theorems developed for standard projection matrices, these methods require that the number of IVs be less than the number of observations, and often perform poorly when the number of IVs is close to (but still less than) the number of observations. Recently proposed regularization approaches for inference under many IVs require strong identification or a sparse relationship between the endogenous variables and the IVs to work well. By combining jackknifing with ridge regularization, we provide a test that has the desired asymptotic size under heteroscedasticity, arbitrarily weak identification, and more IVs than observations, all while achieving good power both when the relationship between the endogenous variables and the IVs is sparse and when it is dense.

Throughout this article, we focus on (Anderson and Rubin 1949, henceforth AR) tests. This is because, in addition to being robust to weak IVs, they are also robust to arbitrary relationships between the endogenous variables and the IVs, since they make no assumption on the first-stage projection of the endogenous variables on the IVs. In the context of AR tests with an increasing number of IVs, it is helpful to distinguish three different asymptotic regimes.

The first “moderately many” IVs regime allows the number of IVs, *k_n_*, to grow with the sample size, *n*, but still requires it to be asymptotically negligible with respect to the sample size. Examples include Andrews and Stock (2007), who require $k_{n}/n^{1/3}\to 0$, and Phillips and Gao (2017), who require $k_{n}/n\to 0$.

The second “many” IVs regime allows the number of IVs to be of the same order of magnitude as the number of observations. Anatolyev and Gospodinov (2011) provide an AR test that remains valid for $k_{n}/n\to \tau ,0\leq \tau <1,n\to \infty$, provided that the error terms are homoscedastic and the IVs satisfy a restrictive balanced-design assumption.^2^ Bun, Farbmacher, and Poldermans (2020) provide analogous results for the GMM version of the AR statistic under the assumption of iid data. These results were recently extended in the form of a jackknifed AR statistic by (Crudu, Mellace, and Sándor 2020, henceforth CMS) and (Mikusheva and Sun 2022, henceforth MS) to the case where errors are allowed to display arbitrary heteroscedasticity, and the only assumption on the IVs is that the diagonal entries of the projection matrix of the IVs are bounded away from unity from above.^3^

The third “very many” IVs regime allows the number of IVs to grow with *n*, and further allows the number of IVs to be greater than the number of observations. (Belloni et al. 2012, henceforth BCCH) propose a Sup Score test that remains valid under mild conditions, and allows the number of IVs to increase exponentially with the sample size. (Carrasco and Tchuente 2016, henceforth CT) propose a ridge-regularized AR statistic that allows for more IVs than observations under the assumption of iid data and homoscedasticity. Kapetanios, Khalaf, and Marcellino (2015) extend Bai and Ng (2010) by proposing weak-identification robust factor-based tests that in principle allow for the number of IVs to be larger than the number of observations, provided that the factor structure of the IVs is sufficiently strong.

Using the notation of the model introduced in the next section, Table 1 provides a schematic overview of the main assumptions and results in the literature on inference that is robust to many weak IVs.

**Table 1:** Anderson and Rubin (1949) tests with many IVs: schematic comparison of main assumptions and results in the literature.

	*k_n_*	*Z*	*ε*
Kaffo and Wang (2017) and Anatolyev and Gospodinov (2011)	$k_{n}/n\to \tau ,0\leq \tau <1,n\to \infty$	*Z* is fixed, obeys restrictive balanced design assumption	iid, $E[\varepsilon_{i}]=0,E[\varepsilon_{i}^{4}]<\infty$
Belloni et al. (2012)	$\;\log\;(\max(k_{n},n))=o(n^{1/3}),n\to \infty$	*Z* is fixed	independent, $E[\varepsilon_{i}]=0,E[\varepsilon_{i}^{3}]<\infty$
Kapetanios, Khalaf, and Marcellino (2015)	Factor-dependent	Potentially dependent data, $E[Z_{il}^{4}]<\infty ,l=1,\ldots ,k_{n},E[\varepsilon_{i}^{4}]<\infty$	
Carrasco and Tchuente (2016)	n→∞	$Z_{i}\varepsilon_{i}$ is iid, $E[Z_{i}\varepsilon_{i}]=0,E[X_{ig}^{2}]<\infty$	
Bun, Farbmacher, and Poldermans (2020)	$k_{n}/n\to \tau ,0\leq \tau <1,n\to \infty$	$Z_{i}\varepsilon_{i}$ is iid, *Z* obeys restrictive balanced design assumption, $E[Z_{i}\varepsilon_{i}]=0,E[Z_{i}^{8}\varepsilon_{i}^{8}]<\infty$	
Crudu, Mellace, and Sándor (2020)	$k_{n}/n\to \tau ,0\leq \tau <1,k_{n}\to \infty$ as n→∞	*Z* is fixed, $P_{ii}\leq 1-\delta ,0<\delta <1$	independent, $E[\varepsilon_{i}]=0,E[\varepsilon_{i}^{4}]<\infty$
Mikusheva and Sun (2022)	$k_{n}/n\to \tau ,0\leq \tau <1,k_{n}\to \infty$ as n→∞	*Z* is fixed, $P_{ii}\leq 1-\delta ,0<\delta <1$	independent, $E[\varepsilon_{i}]=0,E[\varepsilon_{i}^{6}]<\infty$
RJAR	$r_{n}\to \infty$ as n→∞	*Z* is fixed, $\underset{n\to \infty }{\lim\inf}\frac{1}{r_{n}}\sum_{i=1}^{n}\sum_{j\neq i}{\left(P_{ij}^{\gamma_{n}}\right)}^{2}>0$	independent, $E[\varepsilon_{i}]=0,E[\varepsilon_{i}^{4}]<\infty$

NOTE: *n* is the number of observations, *k_n_* is the number of IVs, and *g* is the number of endogenous variables. *Z* is the $n\times k_{n}$ matrix of IVs. *X* is the *n* × *g* matrix of endogenous variables. *ε* is the n×1 vector of structural error terms.

$r_{n}:=\text{rank}(Z)$. See also (1).

$P:=Z{\left(Z\prime Z\right)}^{-1}Z\prime ,P^{\gamma_{n}}:=Z{\left(Z\prime Z+\gamma_{n}I_{k_{n}}\right)}^{-1}Z\prime$ for $\gamma_{n}\geq 0$ if *r_n_* = *k_n_* and $\gamma_{n}\geq \gamma_{-}>0$ if *r_n_* < *k_n_*.

Our test provides a twofold extension of the existing literature. First, it allows for valid inference under many IVs and heteroscedastic errors while further weakening the assumptions of similar tests proposed by CMS and MS. This is made possible by deriving the limiting behavior of the RJAR statistic from the bottom up, without relying on the existing results in Chao et al. (2012) or Hansen and Kozbur (2014). Second, this test allows for more IVs than observations under arbitrary heteroscedasticity of the error terms. The only other approach currently available in the literature that is robust to heteroscedastic error terms and more IVs than observations is the Sup Score test of BCCH. Simulations show that the RJAR test has power comparable to the Sup Score test of BCCH whenever the signal in the first stage is sparse (i.e., only a few of the IVs are informative), and substantially more power when the signal in the first stage is dense (i.e., not sparse). Simulations also show that the RJAR test is as powerful as, and in some cases more powerful than, the AR test of CT, the validity of which has been established only under homoscedastic error terms. Finally, we provide a comparison of the most recently proposed approaches to conducting inference in possibly heteroscedastic linear IV models when the number of IVs is not negligible with respect to the sample size. Indeed, using extensive simulation evidence and an empirical application based on Card (2009), we provide a comparison between these existing “state-of-the-art” methods in a controlled and comparable setting.

In the rest of the article, we use the following notation. *I_p_* denotes the *p* × *p* identity matrix. For an m×1 vector $a=[a_{1},\ldots ,a_{m}]\prime ,||a|{|}_{2}:=\sqrt{\sum_{j=1}^{m}a_{j}^{2}}$. The entry (*i*, *j*) for an *m* × *p* matrix *A* is denoted as *A_ij_* for 1≤i≤m,1≤j≤p. $\text{tr}(A):=\sum_{i=1}^{m}A_{ii}$ for any *m* × *m* matrix *A* with entries given by *A_ij_* for 1≤i,j≤m, and $||A|{|}_{F}:=\sqrt{\text{tr}(A\prime A)}$ denotes the Frobenius norm of *A*. The remaining notation follows standard conventions.

The structure of the article is as follows. Section 2 specifies the linear IV model considered throughout. Section 3 introduces the RJAR test, and provides the main asymptotic results. Section 4 provides simulation evidence on the size and power of our RJAR test and compares it with the tests proposed by BCCH, CT, CMS, and MS. Section 5 considers an empirical application based on Card (2009). Section 6 concludes. All proofs are given in the supplementary material.

## 2 Model

We consider the heteroscedastic linear IV model given by

(1a) y=Xβ+ε

(1b) X=ZΠ+V,

where *y* is an n×1 vector containing the dependent variable, *X* is an *n* × *g* matrix containing the endogenous variables, *β* is a g×1 coefficient vector, *ε* is an n×1 vector containing the structural error terms, *Z* is an $n\times k_{n}$ matrix containing the IVs, Π is a $k_{n}\times g$ coefficient matrix, and *V* is an *n* × *g* matrix containing the first-stage errors. *k_n_* can diverge with *n*, but *g* is fixed. Also, let *y_i_*, *X_i_*, $\varepsilon_{i}$, *Z_i_*, and *V_i_* denote the *i*th row of y,X,ε,Z, and *V*, respectively. As in BCCH, CMS, and MS, we treat *Z* as fixed (non-stochastic),^4^ and assume that any exogenous covariates have been partialled out (see the discussion of Assumption 3). We *exclusively* consider methods that allow for arbitrarily weak identification, that is, methods that control asymptotic size irrespective of the value of Π.

Inference is conducted on the coefficient vector *β* by testing hypotheses of the following type for a prespecified $\beta_{0}\in R^{g}$:

(2) $$H_{0}:\beta =\beta_{0}\text{ versus }H_{1}:\beta \neq \beta_{0}.$$

For a given non-randomized test of asymptotic size α∈(0,1), a confidence set of asymptotic coverage 1−α can be constructed as the collection of those *β*_0_ for which *H*_0_ in (2) is not rejected. For convenience, define

(3) $$\begin{array}{c} e(\beta_{0}):=\left[e_{1}(\beta_{0}),\ldots ,e_{n}(\beta_{0})\right]\prime ,\\ e_{i}(\beta_{0}):=y_{i}-X_{i}\beta_{0},\;\;i=1,\ldots ,n, \end{array}$$

which we refer to as the structural error under the null hypothesis.

It will become apparent below that so long as the error term, *ε*, remains additive, the linearity in the structural equation (1a) and the first-stage equation (1b) can be relaxed to allow for any (known) real-valued function, without affecting the asymptotic size of the RJAR test.

## 3 The RJAR Test

This section introduces our proposed RJAR test, derives its large sample properties under the null hypothesis in (2), and discusses its relationship to the most closely related tests in the literature: the ridge-regularized AR test of CT, the jackknifed AR tests of CMS and MS, and the Sup Score test of BCCH.

### 3.1 Definition of the RJAR Test

The original AR test of the null hypothesis in (2) can be thought of as a test that the IVs are exogenous using the implied structural errors in (3). More specifically, the AR test is a Wald test of the significance of the IVs in the auxiliary regression of the structural errors under the null, $e_{i}(\beta_{0})$, on the IVs, *Z_i_*.^5^ The weak-IV-robust AR test of the null hypothesis in (2) with asymptotic size *α* rejects the null if and only if the AR statistic exceeds the 1−α quantile of a $\chi^{2}$ distribution with *k_n_* degrees of freedom. When the number of IVs *k_n_* grows with *n*, that is, when there are many (potentially weak) IVs, the $\chi^{2}$ approximation of the original AR statistic breaks down. The recently proposed AR test of CT, which we shall briefly review in Section 3.3, uses ridge-regularization to allow for a growing number of IVs that may even exceed the sample size, but is not robust to arbitrary heteroscedasticity in the error terms. The recent papers by CMS and MS, which we shall also briefly review in Section 3.3, propose jackknifed versions of the AR test that remain valid when *k_n_* grows with *n*, but require $k_{n}<n$. Our proposed method combines ridge regularization with jackknifing of the auxiliary regression of $e_{i}(\beta_{0})$ on *Z_i_* to allow for a weak-identification robust test capable of dealing with both more IVs than observations and arbitrary heteroscedasticity in the error terms.

We first standardize the IVs in-sample (after partialling out any covariates) as in BCCH (p. 2393) so that

(4) $$\frac{1}{n}\sum_{i=1}^{n}Z_{ij}^{2}=1\;\text{for}\;j=1,\ldots ,k_{n}.$$

The RJAR test for the testing problem in (2) is then based on the following statistic:

(5) $$\mathrm{RJA}R_{\gamma_{n}}(\beta_{0}):=\frac{1}{\sqrt{r_{n}}\sqrt{{\hat{\Phi }}_{\gamma_{n}}(\beta_{0})}}\sum_{i=1}^{n}\sum_{j\neq i}P_{ij}^{\gamma_{n}}e_{i}(\beta_{0})e_{j}(\beta_{0}),$$

where $r_{n}:=\text{rank}(Z)$ (assumed to be positive without loss of generality),

(6) $${\hat{\Phi }}_{\gamma_{n}}(\beta_{0}):=\frac{2}{r_{n}}\sum_{i=1}^{n}\sum_{j\neq i}{\left(P_{ij}^{\gamma_{n}}\right)}^{2}e_{i}^{2}(\beta_{0})e_{j}^{2}(\beta_{0}),$$

and $P^{\gamma_{n}}:=Z{\left(Z\prime Z+\gamma_{n}I_{k_{n}}\right)}^{-1}Z\prime$ is the ridge-regularized projection matrix with $\gamma_{n}\geq 0$ if *r_n_* = *k_n_* and $\gamma_{n}>0$ if *r_n_* < *k_n_ . γ_n_* is a (sequence of) regularization parameter(s) whose choice we discuss below. We note that setting $\gamma_{n}=0$ when *r_n_* = *k_n_* corresponds to the case where a standard least-squares projection matrix $Z{\left(Z\prime Z\right)}^{-1}Z\prime$ is used to construct the RJAR statistic. This is the standard jackknife statistic. We also note that the *r_n_*-scaling in the denominator of the RJAR statistic cancels with the *r_n_* in the denominator of ${\hat{\Phi }}_{\gamma_{n}}(\beta_{0})$.

We set the regularization parameter *γ_n_* to

(7) $$\gamma_{n}^{*}:=\max\underset{\gamma_{n}\in \Gamma_{n}}{\arg\max}\sum_{i=1}^{n}\sum_{j\neq i}{\left(P_{ij}^{\gamma_{n}}\right)}^{2},$$

where $\Gamma_{n}:=\{\gamma_{n}\in R:\gamma_{n}\geq 0\text{ if }r_{n}=k_{n},\text{ and }\gamma_{n}\geq \gamma_{-}>0\text{ if }r_{n}<k_{n}\}$ for some constant $\gamma_{-}$ not depending on *n*. The existence of $\gamma_{n}^{*}$ is shown in the supplementary material. We let $\gamma_{n}^{*}$ be an element of $\underset{\gamma_{n}\in \Gamma_{n}}{\arg\max}\sum_{i=1}^{n}\sum_{j\neq i}{\left(P_{ij}^{\gamma_{n}}\right)}^{2}$ out of conservativeness, that is, to make Assumption 3 as plausible as possible given the IVs. Furthermore, we let $\gamma_{n}^{*}$ be the maximal element of this set because the maximizer is not necessarily unique without imposing additional assumptions on the singular values and left-singular vectors of the IVs (although in practice we only found unique maximizers). We choose to take the maximum of the maximizers to make the smallest eigenvalues of the ridge-regularized Gram matrix, $Z\prime Z+\gamma_{n}I_{k_{n}}$, as far away from zero as possible when *r_n_* < *k_n_*. To see that $\gamma_{n}^{*}$ maximizes the smallest eigenvalue of $Z\prime Z+\gamma_{n}I_{k_{n}}$ among the elements of $\underset{\gamma_{n}\in \Gamma_{n}}{\arg\max}\sum_{i=1}^{n}\sum_{j\neq i}{\left(P_{ij}^{\gamma_{n}}\right)}^{2}$, notice that due to the symmetry of $Z\prime Z+\gamma_{n}I_{k_{n}}$, its smallest eigenvalue can be expressed as

$$\underset{a\in R^{k_{n}}:||a|{|}_{2}=1}{\text{ min }}a\prime (Z\prime Z+\gamma_{n}I_{k_{n}})a=\underset{a\in R^{k_{n}}:||a|{|}_{2}=1}{\text{ min }}a\prime (Z\prime Z)a+\gamma_{n}=\gamma_{n},$$

where the first equality used that a′a=1, and the second equality used that the smallest eigenvalue of Z′Z is 0 when the rank *r_n_* of *Z* is less than *k_n_*.

We now have all the ingredients to define our new test.

Definition 3.1. The RJAR test rejects $H_{0}:\beta =\beta_{0}$ in (2) at significance level α∈(0,1) if and only if

(8) $$\mathrm{RJA}R_{\gamma_{n}^{*}}(\beta_{0})>Q(1-\alpha ),$$

where $\mathrm{RJA}R_{\gamma_{n}}(\beta_{0})$ is defined in (5), $\gamma_{n}^{*}$ is defined in (7), and Q(1−α) is the (1−α) quantile of the Standard Normal distribution.

### 3.2 Asymptotic Properties of the RJAR Test

We make the following assumptions to derive the limiting distribution of $\mathrm{RJA}R_{\gamma_{n}^{*}}(\beta_{0})$ under the null hypothesis in (2).

Assumption 1. ${\left\{\varepsilon_{i}\right\}}_{i\in N}$ is a sequence of independent random variables satisfying $E[\varepsilon_{i}]=0,\underset{i\in N}{\inf}\text{var}[\varepsilon_{i}]>0$, and $\underset{i\in N}{\sup}E[\varepsilon_{i}^{4}]<\infty$.

Assumption 2. $r_{n}=\text{rank}(Z)\to \infty$ as n→∞.

Assumption 3.

1. If *r_n_* = *k_n_*, then there exists a $\gamma_{n}\in [0,\infty )$ such that $\underset{n\to \infty }{\lim\inf}\frac{1}{r_{n}}\sum_{i=1}^{n}\sum_{j\neq i}{\left(P_{ij}^{\gamma_{n}}\right)}^{2}>0$.
2. If *r_n_* < *k_n_*, then there exists a $\gamma_{-}>0$ and a $\gamma_{n}\in [\gamma_{-},\infty )$ such that
  $$\underset{n\to \infty }{\lim\inf}\frac{1}{r_{n}}\sum_{i=1}^{n}\sum_{j\neq i}{\left(P_{ij}^{\gamma_{n}}\right)}^{2}>0.$$

Assumption 1 is a mild condition on the structural error terms, and allows for conditional heteroscedasticity. It is the same as in CMS. It is slightly less restrictive than the one in MS (who require finite sixth moments on the structural error terms), and slightly more restrictive than the one in BCCH (who require finite third moments).^6^

Assumption 2 is a weak technical assumption. It implies that both *k_n_* and *n* diverge. It also allows for the sum of the number of IVs and the number of exogenous covariates to be larger than the number of observations, provided that the number of exogenous covariates that have been partialled out be sufficiently small (so that the rank of the matrix of partialed IVs continues to diverge).^7^ This assumption is weaker than the restriction on the dimensionality in MS and CMS, who require *r_n_* = *k_n_*, and $k_{n}<n$ for each n∈N, as well as $k_{n}\to \infty$ as n→∞. BCCH prove asymptotic size control of their Sup Score test under the assumption that $\;\log\;k_{n}=o(n^{1/3})$.

#### 3.2.1 Assumption 3 when *r_n_* = *k_n_*

Assumption 3 is a high-level assumption on the number of IVs and their correlation structure. Assumption 3 implies that $\gamma_{n}^{*}$ satisfies $\underset{n\to \infty }{\lim\inf}\frac{1}{r_{n}}\sum_{i=1}^{n}\sum_{j\neq i}{\left(P_{ij}^{\gamma_{n}^{*}}\right)}^{2}>0$. In the absence of exogenous covariates and when $k_{n}<n$ (as in CMS and MS), Assumption 3 is weaker than the *balanced-design* assumption in CMS and MS which requires for $P:=Z{\left(Z\prime Z\right)}^{-1}Z\prime$ that

(9) $$\underset{1\leq i\leq n}{\text{ max }}P_{ii}\leq 1-\delta \;\;\text{for all }n\in N,$$

for some 0<δ<1. This is stated formally in part 1 of the following Proposition.

Proposition 3.1. Suppose (as in CMS and MS) that $\text{rank}(P)=k_{n}$.

1. If there exists a δ∈(0,1) such that $\underset{1\leq i\leq n}{\text{ max }}P_{ii}\leq 1-\delta$ for all n∈N then Assumption 3 is satisfied.
2. For any δ∈(0,1), let $A_{n}(\delta ):=\{i\in \{1,\ldots ,n\}:P_{ii}\geq 1-\delta \}$. If there exists a δ′∈(0,1) such that $\frac{1}{k_{n}}|A_{n}(\delta \prime )|\to 0$ then Assumption 3 is satisfied.

Part 2. of Proposition 3.1 implies that Assumption 3 can be satisfied—even when the balanced-design assumption is not—as long as the number of diagonal elements of *P* “close to 1” increases slower than *k_n_*. Note also that if for some δ′∈(0,1) it holds that $\underset{1\leq i\leq n}{\text{ max }}P_{ii}\leq 1-\delta \prime$ for all n∈N, then $A_{n}(\delta \prime )=\emptyset$. Thus, part 1. of Proposition 3.1 actually follows from Part 2. However, we have chosen to keep part 1. as a separate statement to explicitly state that the balanced-design assumption implies Assumption 3.

#### 3.2.2 Assumption 3 When *r_n_* < *k_n_*

When the number of instruments exceeds the sample size, that is when $k_{n}>n$, one has that $r_{n}\leq n$. The following proposition provides sufficient conditions for Assumption 3 to be satisfied with probability one in this situation in case *Z* is random.

Proposition 3.2. Let the entries of *Z* be iid with a mean zero and variance one. In addition, let *p* > 2 satisfy $E|Z_{11}{|}^{4p}<\infty$ and let $k_{n}=\tau n$ for τ∈[1,∞). Then, if the distribution of *Z*_11_ is absolutely continuous with respect to the Lebesgue measure, there exists a constant η>0 such that for $\gamma_{n,\eta }=\eta n$ it holds with probability one that

$$\underset{n\to \infty }{\lim\inf}\frac{1}{r_{n}}\sum_{i=1}^{n}\sum_{j\neq i}{\left(P_{ij}^{\gamma_{n,\eta }}\right)}^{2}>0.$$

Note that Assumption 3 is satisfied in particular when the entries of *Z* are iid Gaussian. The latter was used in Hansen and Kozbur (2014) to provide a set of sufficient conditions for their assumptions to be satisfied. The proof of Proposition 3.2 also sheds further light on the exact value of *η*. To summarize, Propositions 3.1 and 3.2 show that Assumption 3 is likely to be satisfied; this can be the case even in settings in which the assumptions underlying previous methods are violated.

If very small values of $\frac{1}{r_{n}}\sum_{i=1}^{n}\sum_{j\neq i}{\left(P_{ij}^{\gamma_{n}}\right)}^{2}$ are observed, then Assumption 3 may be questionable. If this is the case, practitioners could make Assumption 3 more plausible by suitably modifying the matrix of IVs, *Z*. This could include the dropping of IVs (either randomly or motivated by economic reasoning, but not informed by the in-sample correlation of the IVs with the endogenous variable), or the (iterative) removal of certain observations. If sufficient data is available to split the sample, a data-driven selection of IVs on one split of the sample for use on the other is also possible.

#### 3.2.3 Asymptotic Normality of the RJAR Test Statistic

We are now in a position to state the asymptotic distribution of the RJAR test under the null hypothesis.

Theorem 3.1. Suppose Assumptions 1–3 and the null hypothesis in (2) hold. Consider any sequence *γ_n_* such that $\underset{n\to \infty }{\lim\inf}\frac{1}{r_{n}}\sum_{i=1}^{n}\sum_{j\neq i}{\left(P_{ij}^{\gamma_{n}}\right)}^{2}>0$. Then, the statistic $\mathrm{RJA}R_{\gamma_{n}}(\beta_{0})$ defined in (5) satisfies

$$\mathrm{RJA}R_{\gamma_{n}}(\beta_{0})\overset{d}{\to }N[0,1].$$

Corollary 3.1. Under Assumptions 1–3 and the null hypothesis in (2), the RJAR test given in Definition 3.1 (i.e., using $\gamma_{n}=\gamma_{n}^{*}$ for all n∈N) has asymptotic size *α*.

Notice that we did not impose any assumption on the coefficients of the instruments Π in the first-stage regression in (1). Therefore, the RJAR test is robust to arbitrarily weak identification.

### 3.3 Closest Alternatives in the Literature

The RJAR test combines two existing approaches in the literature. It uses ridge-regularization as in CT to allow for *r_n_* < *k_n_*, and jackknifing as in CMS and MS to allow for arbitrary heteroscedasticty in the error terms.

The RJAR test is similar to the ridge-regularized AR test proposed by CT given by

$$AR_{CT}=\frac{ne(\beta_{0})\prime P^{\theta }e(\beta_{0})}{e(\beta_{0})\prime (I_{n}-P^{\theta })e(\beta_{0})},$$

where $P^{\theta }=Z{\left(Z\prime Z+\theta I_{k}\right)}^{-1}$ for some fixed scalar *θ* that does not depend on *n*, where θ≥0 if *r_n_* = *k_n_*, and θ>0 if *r_n_* < *k_n_*. CT show that under the assumption of homoscedastic error terms, *AR_CT_* converges to an infinite sum of weighted $\chi_{1}^{2}$ distributions. Since the limiting distribution depends on an infinite number of unobserved weights, CT propose a bootstrap procedure to derive the critical values for *AR_CT_*. The distinguishing features of the RJAR test are the data-driven and unique penalty parameter used to regularize the projection matrix (we find substantial sensitivity of the performance of *AR_CT_* to different values of *θ*), the robustness to arbitrary heteroscedasticiy in the error terms, and its computational speed (it uses standard Normal asymptotic critical values instead of the bootstrap).

Robustness to arbitrary heteroscedasticity in the error terms is achieved through jackknifing, as in CMS and MS. The distinguishing feature of the RJAR test to the tests proposed in CMS and MS is the use of a ridge-regularized projection matrix $P^{\gamma_{n}}$, which makes the RJAR test applicable also when *k_n_* > *r_n_*, unlike the aforementioned two tests that use the standard least-squares projection matrix $Z\prime {\left(Z\prime Z\right)}^{-1}Z\prime$, and cannot be computed when *k_n_* > *r_n_*.

CMS assume $r_{n}=k_{n}<n$, and propose the jackknifed AR statistic given by

$$AR_{\mathrm{CMS}}(\beta_{0}):=\frac{1}{\sqrt{k_{n}}\sqrt{{\hat{\Phi }}_{\mathrm{CMS}}(\beta_{0})}}\sum_{i=1}^{n}\sum_{j\neq i}C_{ij}e_{i}(\beta_{0})e_{j}(\beta_{0}),$$

where $C:=A-B,A:=P+\Delta ,B:=(I_{n}-P)D{\left(I_{n}-D\right)}^{-1}(I_{n}-P),\Delta :=PD{\left(I_{n}-D\right)}^{-1}P-\frac{1}{2}PD{\left(I_{n}-D\right)}^{-1}-\frac{1}{2}D{\left(I_{n}-D\right)}^{-1}P$, and *D* is the diagonal matrix containing the diagonal elements of *P*. ${\hat{\Phi }}_{\mathrm{CMS}}(\beta_{0}):=\frac{2}{k_{n}}\sum_{i=1}^{n}\sum_{j\neq i}C_{ij}^{2}e_{i}^{2}(\beta_{0})e_{j}^{2}(\beta_{0})$. Under the null hypothesis in (2), CMS show that $AR_{\mathrm{CMS}}(\beta_{0})$ converges to a Standard Normal distribution. The CLT underlying this result is a modified version of Lemma A2 of Chao et al. (2012) proposed in (Bekker and Crudu 2015, Appendix A.4).

MS also assume that $r_{n}=k_{n}<n$, and propose a different jackknifed AR statistic than CMS that can be obtained from $\mathrm{RJA}R_{\gamma_{n}}(\beta_{0})$ in (5) by setting $\gamma_{n}=0$, and replacing ${\hat{\Phi }}_{\gamma_{n}}(\beta_{0})$ with

(10) $${\hat{\Phi }}_{MS}(\beta_{0}):=\frac{2}{k_{n}}\sum_{i=1}^{n}\sum_{j\neq i}\frac{P_{ij}^{2}}{M_{ii}M_{jj}+M_{ij}^{2}}\left[e_{i}(\beta_{0})M_{i}e(\beta_{0})\right]\left[e_{j}(\beta_{0})M_{j}e(\beta_{0})\right],$$

where $M=I_{n}-P$, and *M_i_* is the *i*th row of *M*. The reason why the unregularized jackknifed AR test in MS uses ${\hat{\Phi }}_{MS}(\beta_{0})$ instead of the variance estimator given in (6) evaluated at $\gamma_{n}=0$, is because, according to their Theorem 4, and the discussion in Section 4.2.1, the former yields higher power than the latter. It follows that the unregularized version of our RJAR test, which arises when $\gamma_{n}^{*}=0$ in (7), will be dominated by the jackknifed AR test of MS in terms of power, and practitioners may prefer the latter when *r_n_* = *k_n_* and the balanced-design assumption is satisfied. This can have implications for the finite-sample performance of the jackknifed AR test of MS compared to the RJAR, which we investigate in Section 4.

The only other existing test that allows for *r_n_* < *k_n_* and arbitrary heteroscedasticty is the Sup Score test of BCCH. BCCH first standardize the IVs as in (4), and then propose the Sup Score statistic given by

$$S(\beta_{0})=\underset{1\leq j\leq k_{n}}{\text{ max }}\frac{\left|\frac{1}{\sqrt{n}}\sum_{i=1}^{n}e_{i}(\beta_{0})Z_{ij}\right|}{\sqrt{\frac{1}{n}\sum_{i=1}^{n}{\left(e_{i}(\beta_{0})\right)}^{2}Z_{ij}^{2}}}.$$

BCCH propose to use the critical value $\nu_{\alpha }=c_{\mathrm{BCCH}}Q\left(1-\alpha /(2k_{n})\right)$, *c_BCCH_* > 1 and α∈(0,1). They show that comparing their Sup Score statistic to this critical value yields a test of the null hypothesis in (2) that has asymptotic size less than or equal to *α*. Being a supremum-norm test suggests that the BCCH Sup Score test will work well with a sparse first stage (i.e., where only a few elements of Π are zero), but may have lower power than the RJAR test when the first stage is dense. This is verified in the simulations in Section 4.

## 4 Simulations

We now investigate the size and power properties of the RJAR test and compare them to those of the tests proposed in CT, CMS, MS, and BCCH. We take our simulation setup from Hansen and Kozbur (2014) who in turn take theirs from BCCH. The DGP is given by

(11a) $$y_{i}=X_{i}\beta +\varepsilon_{i}$$

(11b) $$X_{i}=Z_{i}^{\prime }\pi +v_{i},$$

for i=1,…,n=100. The IVs *Z_i_* are independent and identically Gaussian with mean 0 and $\text{var}\left[Z_{il}\right]=0.3$ and $\text{corr}\left[Z_{il},Z_{im}\right]=0.5^{\left|l-m\right|}$.^8^ The error terms are given by

$$\begin{array}{cc} \left[\begin{array}{c} \varepsilon_{i}\\ v_{i} \end{array}\right] & ∼N\left[0,\left[\begin{array}{cc} \sigma_{\varepsilon }^{2} & \sigma_{\varepsilon v}\\ \sigma_{\varepsilon v} & \sigma_{v}^{2} \end{array}\right]\right] \end{array}$$

with $\sigma_{\varepsilon }^{2}=2,\sigma_{v}^{2}=1$ and $\sigma_{\varepsilon v}=0.6\sigma_{\varepsilon }\sigma_{v}$.

π=ζκ, where *κ* is a vector of zeros and ones that varies with the type of DGP considered (sparse or dense, as modeled below), and *ζ* is some scalar that ensures that for a given concentration parameter, $\mu^{2}$, the following relationship is satisfied:

$$\mu^{2}=\frac{n\pi \prime E\left[Z_{i}Z_{i}^{\prime }\right]\pi }{\sigma_{v}^{2}}.$$

This implies that

$$\zeta =\sqrt{\frac{\sigma_{v}^{2}\mu^{2}}{n\kappa \prime E\left[Z_{i}Z_{i}^{\prime }\right]\kappa }}.$$

To illustrate how the sparsity structure of the first stage in (11b) can affect the size and power of the studied tests, we consider both a sparse first stage and a dense first stage. Sparsity in the first stage is modeled by setting $\kappa =[\iota_{5}^{\prime },0_{k_{n}-5}\prime ]\prime$, where *ι_q_* is a q×1 vector of ones, and $0_{q}$ is a q×1 vector of zeros. Denseness in the first stage is modeled by setting $\kappa =[\iota_{0.4k_{n}}\prime ,0_{0.6k_{n}}\prime ]\prime$.

We consider $k_{n}=30,90,190$. In the context of Assumption 3, we search over values greater than 1 when choosing $\gamma_{n}^{*}$ in case *r_n_* < *k_n_*. For the case of 30 IVs, the RJAR test does not impose any regularization ($\gamma_{n}^{*}=0$). For the case of 90 IVs, $\gamma_{n}^{*}=12.048$. We note that in the latter case $\sum_{i=1}^{n}\sum_{j\neq i}{\left(P_{ij}^{\gamma_{n}^{*}}\right)}^{2}=11.123>8.845=\sum_{i=1}^{n}\sum_{j\neq i}{\left(P_{ij}\right)}^{2}$. This shows that even in the case where $r_{n}<n$, ridge regularization can make Assumption 3 strictly more plausible. For the case of 190 IVs, $\gamma_{n}^{*}=109.187$.

The variance estimator of MS occasionally yields a negative value. These cases are conservatively interpreted as a failure to reject the null hypothesis. As recommended by BCCH, *c_BCCH_* = 1.1. As in the simulation section in CT, we set θ=0.05. The number of Monte Carlo replications is 10,000.

The supplementary material provides additional simulation evidence on inference with heteroscedastic error terms. The supplementary material provides additional simulation evidence on projection-based inference.

### 4.1 Size

**Fig. 1:**
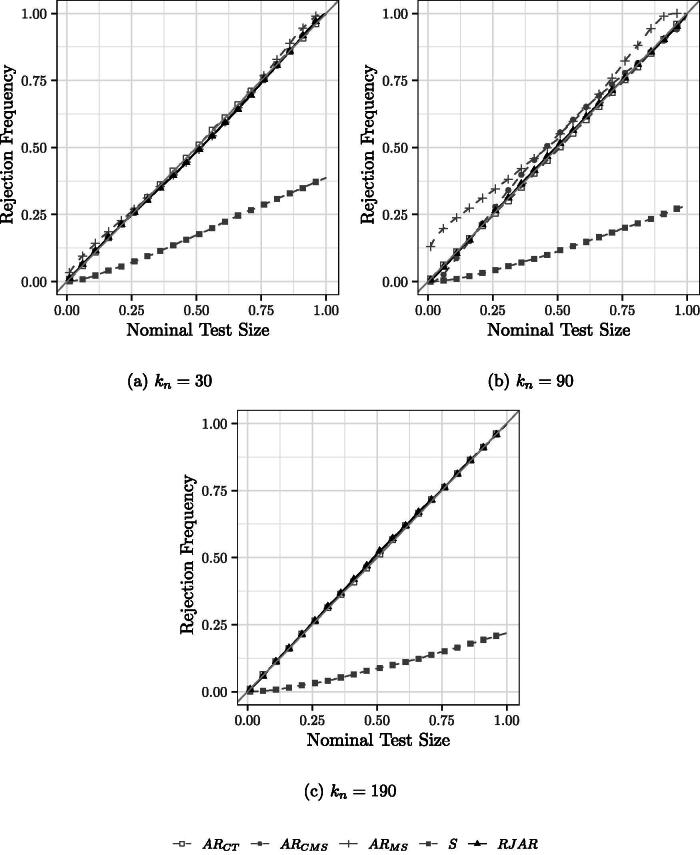
PP Plots for Sparse IVs, homoscedastic errors, *β* = 1, $\mu^{2}=0,H_{0}:\beta_{0}=1$.

Figure 1 shows the simulation results with a sparse first stage for tests of size 0.01 to 0.99, that is the rejection frequency under $H_{0}:\beta_{0}=1$. As far as the illustration of the tests’ size properties is concerned, the dense first stage yields virtually the same results, and is hence omitted. Since all tests are robust to weak IVs, the rejection frequencies of the tests are not affected by the strength of identification. For the case of 30 IVs, the AR tests of CT, CMS, and MS and the RJAR test have correct size, while the BCCH Sup Score test is undersized.

For the case of 90 IVs, the AR test of CT and the RJAR test control size. The AR test of CMS appears to control size for common small nominal test sizes (e.g., 0.05 or 0.1). The AR test of MS appears to be generally oversized. For example, at nominal level 0.05, the rejection frequency of the test is 0.189.^9^ The BCCH Sup Score test continues to be undersized.

For the case of 190 IVs, only the AR test of CT, the BCCH Sup Score test, and the RJAR test are feasible. As before, the BCCH Sup Score test is undersized, while the AR test of CT and the RJAR test have correct size.

### 4.2 Power

**Fig. 2:**
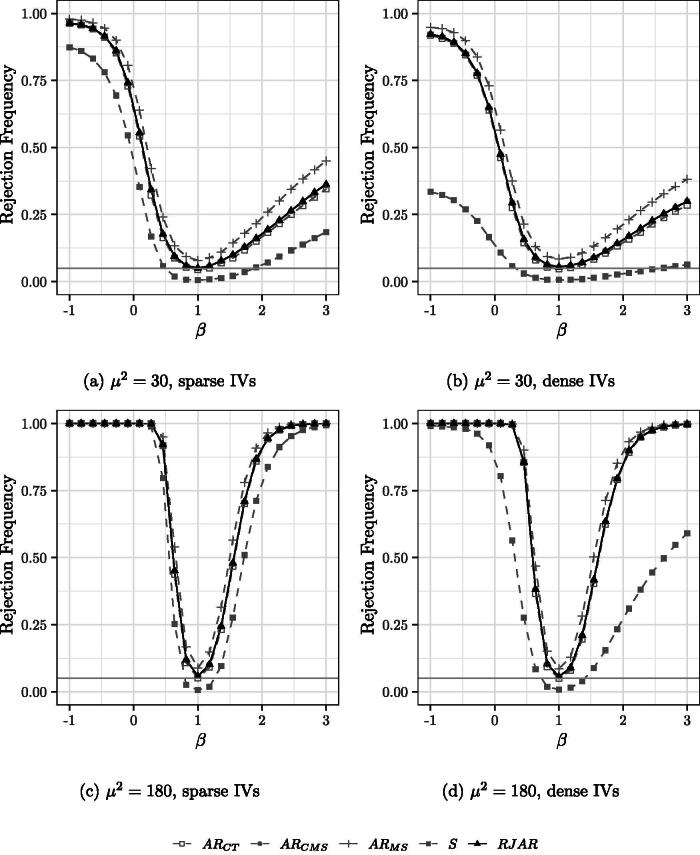
Power curves for 30 IVs. Nominal test size of 5% indicated by the grey horizontal line. $H_{0}:\beta_{0}=1$.

**Fig. 3:**
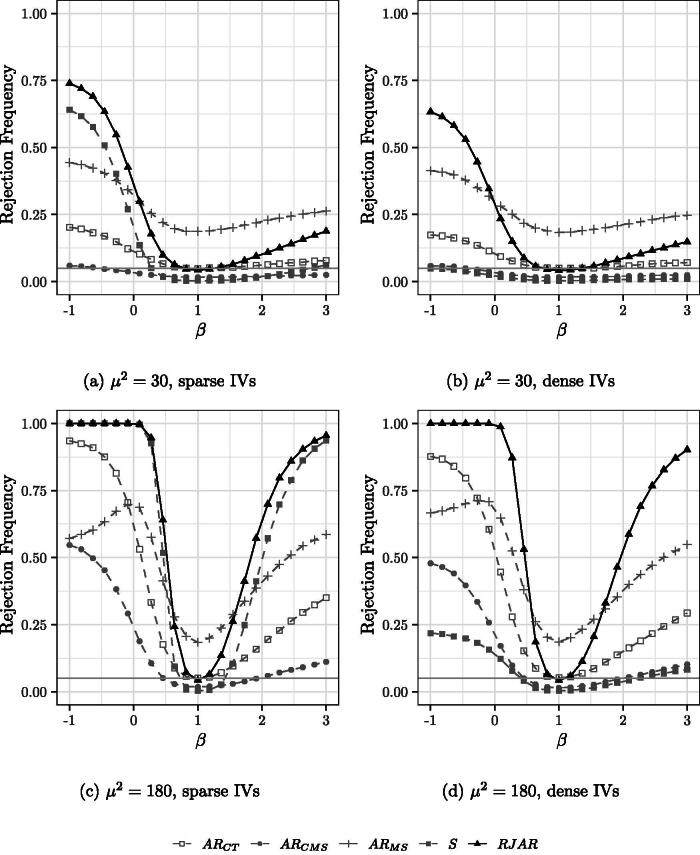
Power curves for 90 IVs. Nominal test size of 5% indicated by the grey horizontal line. $H_{0}:\beta_{0}=1$.

**Fig. 4:**
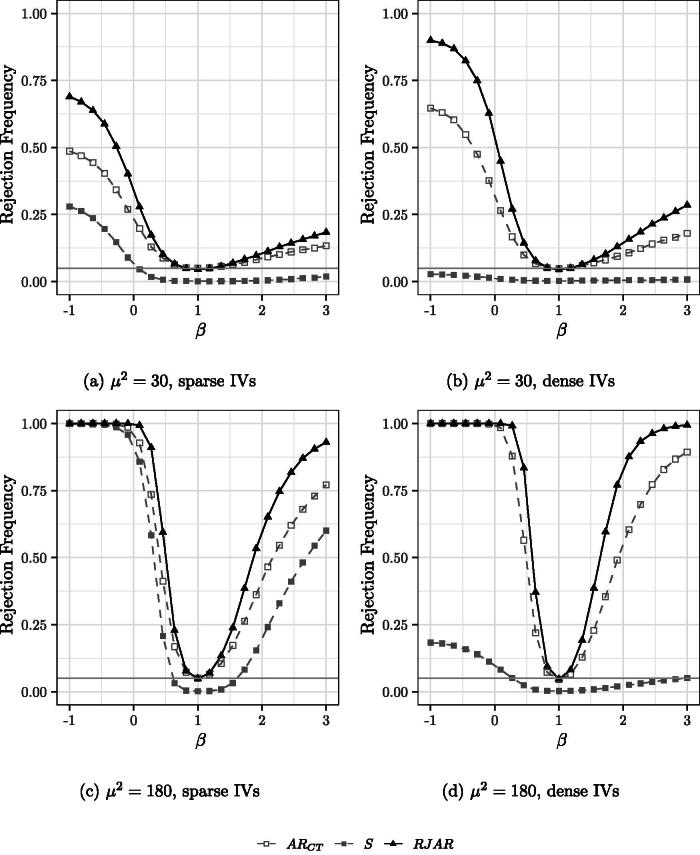
Power curves for 190 IVs. Nominal test size of 5% indicated by the gray horizontal line. $H_{0}:\beta_{0}=1$.

Figures 2–4 show the power of the tests when the number of IVs and the sparsity pattern of the first stage is varied. It is still the case that $H_{0}:\beta_{0}=1$.

For the case of 30 IVs (Figure 2), the AR tests of CT and CMS have similar power to the RJAR test, while MS is slightly more powerful than the RJAR test. The BCCH Sup Score test is less powerful than all other tests.

For the case of 90 sparse IVs (Figure 3), the RJAR test is slightly more powerful than the BCCH Sup Score test. The AR test of MS fails to control the size, while the AR tests of CT and CMS exhibit power properties substantially worse than those of the BCCH Sup Score test and the RJAR test. For the case of 90 dense IVs (Figure 3), the RJAR test is substantially more powerful than all other alternatives. For the case of 190 sparse and dense IVs (Figure 4), the RJAR test is more powerful than the BCCH Sup Score test.^10^ The RJAR test is also more powerful than the AR test of CT. Thus, for all the DGPs that are considered here, the RJAR test is as powerful as existing methods whenever these are applicable, and sometimes much more powerful.

## 5 Empirical Application

We consider an empirical application based on Card (2009). The coefficient of interest is given by *β_s_* in the following model:

(12) $$\begin{array}{cc} y_{is} & =\beta_{s}X_{is}+\delta_{s}^{\prime }W_{i}+\varepsilon_{is}, \end{array}$$

where *y_is_* is the difference between residual log wages for immigrant and native men in skill group *s* in city *i*,^11^ *X_is_* is the log ratio of immigrant to native hours worked in skill group *s* of both men and women in city *i*, and *W_i_* is a vector of city-level controls with coefficient vector *δ_s_*, and $\varepsilon_{is}$ is the structural error. In the context of the production function specified in (Card 2009, sec. I), *β_s_* can be interpreted as the (negative) inverse elasticity of substitution between immigrants and natives in the United States in their respective skill group. As in Card (2009), we consider two skill groups s=h,c (high school or college equivalent) separately.

Card (2009) raises the concern that unobserved factors in a city may lead to both higher wages and higher employment levels of immigrants relative to natives, causing *X_is_* to be endogenous. Card (2009) proposes to use the ratio of the number of immigrants from country *l* in city *i* to the total number of immigrants from foreign country *l* in the United States as an IV. The rationale for these IVs is that existing immigrant enclaves are likely to attract additional immigrant labor through social and cultural channels unrelated to labor market outcomes. We consider two sets of IVs. First, we consider the original setup of Card (2009), using as IVs the *k_n_* = 38 different countries of origin of the immigrants. Second, motivated by the saturation approach of Blandhol et al. (2022), we consider the setup where these 38 original IVs are interacted with the *q* = 9 available controls (including a constant). This yields *k_n_* = 342 IVs. In both cases, the number of observations (i.e., the number of cities) is n=124.^12^

We construct (weak-identification robust) confidence sets for *β_s_* by inverting the AR tests of CT, CMS, and MS, the Sup Score test, and the RJAR test. Thus, the 95% confidence set for any test is obtained as the collection of $\beta_{s,0}$ for which that test does not reject the null at 5% level of significance. As in the simulation exercise in Section 4, we search over values greater than 1 when choosing $\gamma_{n}^{*}$ in case *r_n_* < *k_n_*. Again *c_BCCH_* = 1.1 and θ=0.05. The number of boostrap replications for the AR test of CT is set to 2,500. A grid of 100 values for $\beta_{s,0}$ is used for s=h,c. Data is taken from a single cross section, as made available by Goldsmith-Pinkham, Sorkin, and Swift (2020).

**Fig. 5:**
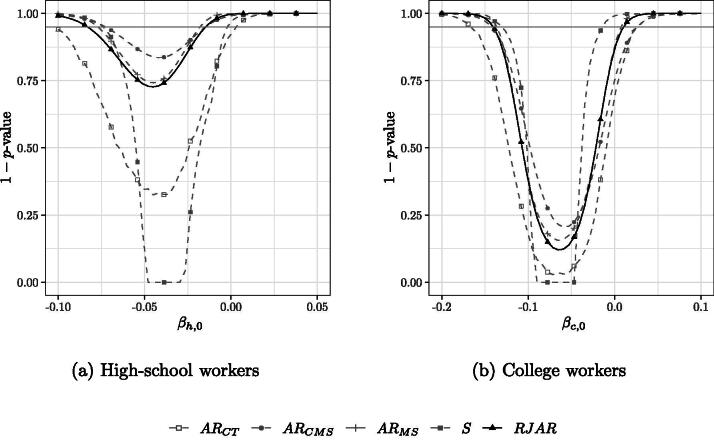
95% confidence sets for *β_s_* for the application in (12) with *k_n_* = 38 IVs. $\max_{i}P_{ii}=0.944$. $\gamma_{n}^{*}=0,r_{n}^{-1}\sum_{i=1}^{n}\sum_{j\neq i}{\left(P_{ij}^{\gamma_{n}^{*}}\right)}^{2}=0.513$.

Figure 5 shows the confidence sets when *k_n_* = 38 for high-school workers and college workers. We find that $\gamma_{n}^{*}=0$, implying that no regularization is needed. This is in line with our simulations in Section 4, where we found that regularization was not needed to maximize the sum in Assumption 3 when $k_{n}/n=0.3$. Furthermore, $\underset{1\leq i\leq n}{\text{ max }}P_{ii}=0.944$, and $r_{n}^{-1}\sum_{i=1}^{n}\sum_{j\neq i}{\left(P_{ij}^{\gamma_{n}^{*}}\right)}^{2}=0.513$. We also point out that only three diagonal entries of *P* are larger than 0.9, which suggests that Assumption 3 is reasonably satisfied by part 2. of Proposition 3.1. The 95% confidence sets for each test are given by all the points below the grey horizontal line. The confidence sets for both skill groups broadly confirm the results in Card (2009). We find that the confidence sets for high-school workers is smallest for the jackknifed AR statistic of CMS, whereas the BCCH Sup Score test yields the smallest confidence interval for the application to college workers. For both cases, the AR test of CT yields the largest confidence interval. Based on the power results in Section 4, this suggests a very sparse first stage for college workers, that is, a few nationalities being highly predictive of inflows of immigrant labor.

**Fig. 6:**
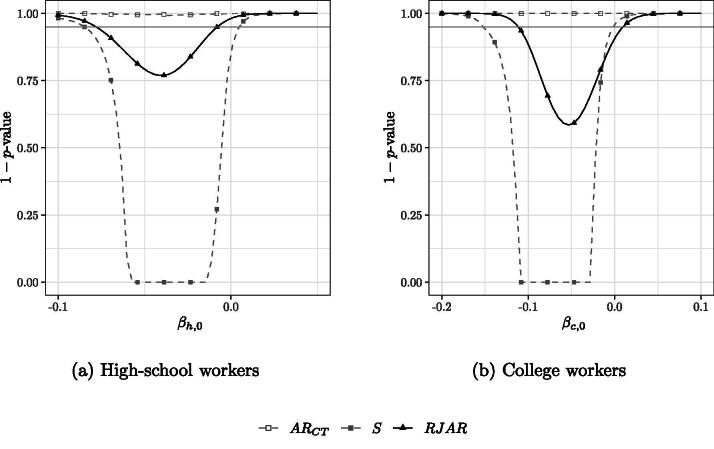
95% confidence sets for *β_s_* for the application in (12) with *k_n_* = 342 IVs. $\gamma_{n}^{*}=5.299,r_{n}^{-1}\sum_{i=1}^{n}\sum_{j\neq i}{\left(P_{ij}^{\gamma_{n}^{*}}\right)}^{2}=0.106$.

Figure 6 shows the confidence sets when $k_{n}=342$ for high-school workers and college workers, respectively. Since $r_{n}=n-q=124-9=115<342=k_{n}$, the jackknifed AR statistics of CMS and MS are not applicable. We find that $\gamma_{n}^{*}=5.299$ and $r_{n}^{-1}\sum_{i=1}^{n}\sum_{j\neq i}{\left(P_{ij}^{\gamma_{n}^{*}}\right)}^{2}=0.106$. The 95% confidence sets obtained by inverting the AR test of CT are empty for both *β_h_* and *β_c_*. This could be due to heteroscedasticity in the error terms. In line with the simulation results on power reported in Section 4, the RJAR test yields smaller confidence intervals than the BCCH Sup Score test. The qualitative conclusions with respect to the case of 38 IVs remain unchanged.

## Conclusion

We contributed to the literature on (very) many IVs in the cross-sectional linear IV model by proposing a new, ridge-regularized jackknifed AR test. Our test compares favorably with existing methods in the literature both theoretically, by allowing for high-dimensional IVs and weakening a common assumption on the IVs’ projection matrix, and practically, by having correct asymptotic size and displaying favorable power properties even when the number of IVs approaches or exceeds the number of observations.

## Supplementary Material

RJARSupplementaryMaterial (2).zip

## Footnotes

1 Recent examples of applications with many IVs include Mueller-Smith (2015), who uses judge dummies interacted with individual covariates as IVs to identify causal effects of incarceration; van Duijn and Rouwendal (2021), who derive an equation for residential sorting that produces many more IVs than observations; and Lönn and Schotman (2022), who derive structural asset-pricing models that lead to a vast number of moment conditions.

2 See Anatolyev and Yaskov (2017) and Crudu, Mellace, and Sándor (2020) for a discussion of the restrictiveness of the balanced-design assumption of Anatolyev and Gospodinov (2011). See also Kaffo and Wang (2017) for a bootstrapped version of the Anatolyev and Gospodinov (2011) AR test.

3 Boot and Ligtenberg (2023), Matsushita and Otsu (2022), and Lim, Wang, and Zhang (2024) propose weak-identification robust jackknife-based procedures for inference with many IVs. However, these methods make additional assumptions on the first-stage projection of the endogenous variables on the IVs, and hence fall outside of the scope of this article.

4 The treatment of IVs as non-stochastic is an assumption that has been commonly used in several of the papers that are most closely related to ours, see Table 1. With stochastic IVs, one can interpret the results as holding conditional on *Z*, see also CMS Footnote 1.

5 The AR statistic is defined as $e(\beta_{0})\prime Z{\hat{\Sigma }}^{-1}Z\prime e(\beta_{0})/n$, where $\hat{\Sigma }$ is a consistent estimator of $\text{var}[Z\prime e(\beta_{0})/\sqrt{n}]$.

6 It is difficult to allow for autocorrelated errors in our framework, since our proof relies on casting the RJAR statistic as a degenerate U-statistic, which requires that $\varepsilon_{i}$ be independent of $\varepsilon_{j}$ for i≠j.

7 More formally, suppose that *W* is an $n\times l_{n}$ matrix of covariates with rank *l_n_*, and define $M_{W}:=I_{n}-W{\left(W\prime W\right)}^{-1}W\prime$. Furthermore, let $\tilde{Z}$ be the $n\times k_{n}$ matrix of IVs prior to partialling out the controls *W*, and let ${\tilde{r}}_{n}:=\text{rank}(\tilde{Z})$, such that $Z=M_{W}\tilde{Z}$ and $r_{n}:=\text{rank}(Z)\leq \min(n-l_{n},{\tilde{r}}_{n})$. Therefore, *l_n_* can diverge as n→∞ without contradicting $r_{n}\to \infty$, which means that a growing number of exogenous controls can be accommodated.

8 Simulations in the supplementary material show that the results remain qualitatively unchanged if uncorrelated Gaussian IVs are considered instead.

9 Simulations in the supplementary material show that if *n* and *k_n_* are increased to about 1000 and 900, respectively, there is virtually no more size distortion.

10 The poor power of the BCCH Sup Score is likely a consequence of the Union Bound underlying their Lemma 5. We note that the poor power properties of the BCCH Sup Score are acknowledged in BCCH, and are what leads them to recommend using their main estimator (Post-LASSO two-stage least squares) whenever weak identification is not a concern, and the first stage is sufficiently sparse.

11 As discussed in (Card 2009, p. 11, footnote 17), residual wages are wages once observed characteristics of the entire U.S. workforce are controlled for.

12 We note that the controls and the IVs are at the city level, and hence the same for the applications to high-school workers and college workers. Therefore, the projection matrix and the ridge-regularized projection matrix will also be the same across these two skill groups.

## Supplementary Materials

The supplementary material contains the proofs of the theoretical results reported in Section 3 of the article and the additional Monte Carlo simulation results mentioned in Section 4 of the article.
