# Supplementary material for: A Ridge-Regularized Jackknifed Anderson-Rubin Test
Source: J Bus Econ Stat. 2023 Dec 12;42(3):1083–94. doi: 10.1080/07350015.2023.2290739 (PMC11182230; doi:10.1080/07350015.2023.2290739)
Supplement: RJARSupplementaryMaterial (2).zip [file UBES_A_2290739_SM3451.zip › RJARSupplementaryMaterial/RJARSupplementaryMaterial/TheoreticalAppendix.pdf]

Supplementary Material

A Ridge-Regularised Jackknifed Anderson-Rubin Test

Max-Sebastian Dovì
International Monetary Fund
and
Anders Bredahl Kock
Department of Economics, University of Oxford and CREATES, Aarhus University
and
Sophocles Mavroeidis
Department of Economics, University of Oxford

November 8, 2023

A Proofs

Throughout this appendix, $C > 0$ denotes a universal constant that can change across lines. CSHNW refers to Chao et al. (2012) from where we also borrow our summation conventions. We also define $\Phi_{\gamma_n} := \frac{2}{r_n}\sum_{i=1}^{n}\sum_{j\neq i}(P_{ij}^{\gamma_n})^2\mathbb{E}[\varepsilon_i^2]\mathbb{E}[\varepsilon_j^2]$. Since all the proofs in this appendix are under the null hypothesis, we write ε_i and $\hat{\Phi}_{\gamma_n}$ for $e_i(\beta_0)$ and $\hat{\Phi}_{\gamma_n}(\beta_0)$, respectively.

Furthermore, we note that Assumption 3 implies that

$$\sum_{i=1}^{n}\sum_{j\neq i}(P_{ij}^{\gamma_n})^2 \geq c r_n, \tag{A.1}$$

for some $c > 0$ and n sufficiently large.

Our strategy for deriving the limiting distribution of our RJAR test is as follows. Since P^{γ_n} is generally not idempotent for $\gamma_n \geq 0$, we cannot rely on standard properties of idempotent matrices. Therefore, we first derive a series of properties for the ridge-regularised projection matrix P^{γ_n} that hold for any $\gamma_n \geq 0$ when $r_n = k_n$ and $\gamma_n > 0$ when $r_n < k_n$. These properties are derived using a singular value decomposition of the matrix of IVs Z, and are collected in Lemmas 1 and 2. These Lemmas play a similar role to Lemma B1 and Lemma B2 in Chao et al. (2014). The proof of Theorem 1 derives the asymptotic distribution of our RJAR statistic under the assumption that Φ_{γ_n} is known. This is achieved by casting the RJAR statistic as a degenerate U statistic with variable kernel, and verifying the conditions of a Martingale CLT. A benefit of deriving our proofs from the bottom up (and not relying on the augmentation approach in Hansen and Kozbur (2014)) is that we do not require that all diagonal elements of P^{γ_n} be bounded away from 1. Lemma 4 shows that our proposed estimator of Φ_{γ_n} is consistent. Taken together with Theorem 1 and Assumption 3, Lemma 4 implies that our RJAR test is asymptotically valid. Finally, Lemma 3 proves the existence of γ_n^*, and the proof of Proposition 1 shows that Assumption 3 is weaker than the balanced-design assumption in CMS and MS.

The following singular value decomposition of the $n \times k_n$ matrix of instruments Z of rank r_n will be used frequently (see, e.g., Lütkepohl (1996, p. 60)):

$$Z = USQ',$$

where U is an $n \times n$ matrix such that $U'U = UU' = I_n$, Q is a $k_n \times k_n$ matrix such that $Q'Q = QQ' = I_{k_n}$, and S is the $n \times k_n$ matrix given by

$$S = \begin{bmatrix} D & 0_{r_n \times (k_n - r_n)} \\ 0_{(n-r_n)\times r_n} & 0_{(n-r_n)\times(k-r_n)} \end{bmatrix},$$

where D is the diagonal $r_n \times r_n$ matrix containing the singular values of Z. Hence, one can write

$$P^{\gamma_n} = USQ'(QS'SQ' + Q\gamma_n I_{k_n} Q')^{-1} QS'U' = US(S'S + \gamma_n I_{k_n})^{-1} S'U' = U\tilde{D}U', \quad \text{(A.2)}$$

where $\tilde{D} = S(S'S + \gamma_n I_{k_n})^{-1}S'$ is the diagonal $n \times n$ matrix with diagonal entries given by given by $\tilde{D}_{ll} = \frac{D_{ll}^2}{D_{ll}^2 + \gamma_n} \leq 1$ for $l = 1, \ldots, r_n$, and zero otherwise. Note that the diagonal entries of $\tilde{D}$ are also the the eigenvalues of P^{γ_n}.

A.1 Lemmas

The following lemma collects some properties of P^{γ_n}. Recall that Z has rank r_n.

Lemma 1. Fix $n \geq 3$. For all $h, m = 1, \ldots, n$ and $\gamma_n \geq 0$ if $r_n = k_n$ and $\gamma_n > 0$ if $r_n < k_n$ one has

(i) $0 \leq (P^{\gamma_n})^j_{hh} \leq P^{\gamma_n}_{hh} \leq 1$ for all positive integers j,

(ii) $\sum_{i=1}^n (P^{\gamma_n}_{hi})^2 = (P^{\gamma_n})^2_{hh} \leq P^{\gamma_n}_{hh}$,

(iii) $\sum_{i=1}^n P^{\gamma_n}_{ii} = \sum_{l=1}^{r_n} \frac{D_{ll}^2}{D_{ll}^2 + \gamma_n} \leq r_n$,

(iv) $\sum_{i=1}^n (P^{\gamma_n})^2_{ii} = \sum_{l=1}^{r_n} \frac{D_{ll}^4}{\left(D_{ll}^2 + \gamma_n\right)^2} \leq r_n$,

(v) $|P^{\gamma_n}_{hm}| \leq 1$,

(vi) For any $\mathcal{I}_2 \subseteq \{1, \ldots, n\}^2$ and any $\mathcal{I}_3 \subseteq \{1, \ldots, n\}^3$,

(a) $\sum_{\mathcal{I}_2} (P^{\gamma_n}_{ij})^4 \leq r_n$,

(b) $\sum_{\mathcal{I}_3} (P^{\gamma_n}_{ij})^2 (P^{\gamma_n}_{jl})^2 \leq r_n$.

Proof. By the arguments prior to this lemma, the non-zero eigenvalues of P^{γ_n} are $\tilde{D}_{ll} = \frac{D^2_{ll}}{D^2_{ll}+\gamma_n}$ for $l = 1, \ldots, r_n$. (iii) now follows from the trace of P^{γ_n} equaling the sum of its non-zero eigenvalues. Furthermore, the eigenvalues of $(P^{\gamma_n})^2$ are given by $\tilde{D}^2_{ll} = \frac{D^4_{ll}}{(D^2_{ll}+\gamma_n)^2}$ for $l = 1, \ldots, r_n$, and zero otherwise. (iv) now follows in the same way as (iii).

By Equation (A.2) one has that

$$(P^{\gamma_n})^j_{hh} = \sum_{l=1}^{n} \tilde{D}^j_{ll} U^2_{hl} \leq \sum_{l=1}^{n} \tilde{D}_{ll} U^2_{hl} = P^{\gamma_n}_{hh} \leq 1, \tag{A.3}$$

which verifies (i) since $0 \leq \tilde{D}_{ll} \leq 1$; the last inequality following from the largest diagonal entry of P^{γ_n} being bounded from above by its largest eigenvalue, which is no greater than one. Next, by (i),

$$\sum_{i=1}^{n} (P^{\gamma_n}_{hi})^2 = (P^{\gamma_n})^2_{hh} \leq P^{\gamma_n}_{hh} \leq 1,$$

such that (ii) and (v) follow. Furthermore, by (v) and (iv),

$$\sum_{\mathcal{I}_2} (P^{\gamma_n}_{ij})^4 \leq \sum_{i=1}^{n} \sum_{j=1}^{n} (P^{\gamma_n}_{ij})^2 = \sum_{i=1}^{n} (P^{\gamma_n})^2_{ii} \leq r_n,$$

and (vi) follows since

$$\sum_{\mathcal{I}_3} (P^{\gamma_n}_{ij})^2 (P^{\gamma_n}_{jl})^2 \leq \sum_{j=1}^{n} \sum_{i=1}^{n} (P^{\gamma_n}_{ij})^2 \sum_{l=1}^{n} (P^{\gamma_n}_{jl})^2 = \sum_{j=1}^{n} \left((P^{\gamma_n})^2_{jj} \right)^2 \leq \sum_{j=1}^{n} (P^{\gamma_n})^2_{jj} \leq r_n,$$

the penultimate inequality being a consequence of (i) and the last of (iv).

□

Lemma 2. Fix $n \geq 4$. For all $\gamma_n \geq 0$ if $r_n = k_n$ and $\gamma_n > 0$ if $r_n < k_n$ one has

$$\left| \sum_{i<j<l<m} P_{il}^{\gamma_n} P_{jl}^{\gamma_n} P_{im}^{\gamma_n} P_{jm}^{\gamma_n} \right| \leq C r_n.$$

Proof. The proof follows Lemma B2 in CSHNW closely. We verify that Lemma B2 in CSHNW remains valid for any symmetric matrix satisfying the properties in Lemma 1. For $G := \operatorname{diag}(P_{11}^{\gamma_n}, \ldots, P_{nn}^{\gamma_n})$ observe that $(P^{\gamma_n} - G)^4$ equals

$$\begin{aligned}
&(P^{\gamma_n})^4 - (P^{\gamma_n})^3 G - (P^{\gamma_n})^2 G P^{\gamma_n} + (P^{\gamma_n})^2 G^2 - P^{\gamma_n} G (P^{\gamma_n})^2 + P^{\gamma_n} G P^{\gamma_n} G \\
&+ P^{\gamma_n} G^2 P^{\gamma_n} - P^{\gamma_n} G^3 - G(P^{\gamma_n})^3 + G(P^{\gamma_n})^2 G + G P^{\gamma_n} G P^{\gamma_n} \\
&- G P^{\gamma_n} G^2 + G^2 (P^{\gamma_n})^2 - G^2 P^{\gamma_n} G - G^3 P^{\gamma_n} + G^4.
\end{aligned}$$

Since $\operatorname{tr}(A') = \operatorname{tr}(A)$ and $\operatorname{tr}(AB) = \operatorname{tr}(BA)$ for square matrices A and B,

$$\begin{aligned}
\operatorname{tr}((P^{\gamma_n} - G)^4) =& \operatorname{tr}((P^{\gamma_n})^4) - 4\operatorname{tr}((P^{\gamma_n})^3 G) + 4\operatorname{tr}((P^{\gamma_n})^2 G^2) + 2\operatorname{tr}(P^{\gamma_n} G P^{\gamma_n} G) \\
& - 4\operatorname{tr}(P^{\gamma_n} G^3) + \operatorname{tr}(G^4) \\
\leq& \operatorname{tr}((P^{\gamma_n})^4) + 4|\operatorname{tr}((P^{\gamma_n})^3 G)| + 4\operatorname{tr}((P^{\gamma_n})^2 G^2) + 2\operatorname{tr}(P^{\gamma_n} G P^{\gamma_n} G) \\
& + 4|\operatorname{tr}(P^{\gamma_n} G^3)| + \operatorname{tr}(G^4).
\end{aligned}$$

Next, because $0 \leq (P^{\gamma_n})_{ii}^j \leq P_{ii}^{\gamma_n}$ for all positive integers j by Lemma 1 (i), and $G_{ii} \geq 0$, one gets

$$\begin{aligned}
\operatorname{tr}((P^{\gamma_n} - G)^4) \leq& \operatorname{tr}(P^{\gamma_n}) + 4\operatorname{tr}(P^{\gamma_n} G) + 4\operatorname{tr}(P^{\gamma_n} G^2) + 2\operatorname{tr}(P^{\gamma_n} G P^{\gamma_n} G) \\
& + 4\operatorname{tr}(P^{\gamma_n} G^3) + \operatorname{tr}(G^4).
\end{aligned}$$

Since $0 \leq P_{ii}^{\gamma_n} \leq 1$ and $G^j \leq I_n$ (elementwise) for all positive integers j, $\operatorname{tr}(P^{\gamma_n} G^j) \leq \operatorname{tr}(P^{\gamma_n}) \leq r_n$, $\operatorname{tr}(P^{\gamma_n} G P^{\gamma_n} G) \leq \operatorname{tr}((P^{\gamma_n})^2) \leq r_n$. Furthermore, $\operatorname{tr}(G^4) \leq \operatorname{tr}(G) = \operatorname{tr}(P^{\gamma_n}) \leq r_n$. Hence,

$$\operatorname{tr}((P^{\gamma_n} - G)^4) \leq 16 r_n. \tag{A.4}$$

As in the proof of Lemma B2 in CSHNW, define the lower-triangular matrix L with entries $L_{ij} = P^{\gamma_n}_{ij}\mathbf{1}_{i>j}$, so that $P^{\gamma_n} = L + L' + G$. Then

$$
\begin{aligned}
(P^{\gamma_n} - G)^4 =& (L + L')^4 \\
=& L^4 + L^2LL' + L^2L'L + L^2L'^2 + LL'L^2 + LL'LL' \\
& + LL'L'L + LL'^3 + L'LL^2 + L'LLL' + L'LL'L + L'LL'^2 \\
& + L'^2L^2 + L'^2LL' + L'^2L'L + L'^4.
\end{aligned}
$$

Note for all positive integers j, $[(L')^j]' = L^j$. Since $\mathrm{tr}(A') = \mathrm{tr}(A)$ and $\mathrm{tr}(AB) = \mathrm{tr}(BA)$ for any square matrices A and B,

$$
\mathrm{tr}((P^{\gamma_n} - G)^4) = 2\mathrm{tr}(L^4) + 8\mathrm{tr}(L^3L') + 4\mathrm{tr}(L^2L'^2) + 2\mathrm{tr}(L'LL'L). \tag{A.5}
$$

We consider each of the terms on the right-hand side of Equation (A.5) separately. Before proceeding, note that:

$$
\begin{aligned}
(L)^2_{ab} &= \sum_{l=1}^{n} L_{al}L_{lb}, \\
(L')^2_{ab} &= \sum_{l=1}^{n} L_{la}L_{bl} = \sum_{l=1}^{n} L_{bl}L_{la} = (L)^2_{ba}, \\
(L'L)_{ab} &= \sum_{l=1}^{n} L_{la}L_{lb}, \\
(L)^3_{ab} &= \sum_{l=1}^{n} (L)^2_{al}L_{lb} = \sum_{l=1}^{n}\sum_{m=1}^{n} L_{am}L_{ml}L_{lb}, \\
(L)^4_{ab} &= \sum_{l=1}^{n} (L)^2_{al}(L)^2_{lb} = \sum_{l=1}^{n}\sum_{m=1}^{n}\sum_{j=1}^{n} L_{am}L_{ml}L_{lj}L_{jb}, \\
(L^3L')_{ab} &= \sum_{l=1}^{n} (L)^3_{al}(L')_{lb} = \sum_{l=1}^{n}\sum_{m=1}^{n}\sum_{j=1}^{n} L_{am}L_{mj}L_{jl}L_{bl}, \\
(L^2L'^2)_{ab} &= \sum_{l=1}^{n} (L)^2_{al}(L')^2_{lb} = \sum_{l=1}^{n} (L)^2_{al}(L)^2_{bl} = \sum_{l=1}^{n}\sum_{m=1}^{n}\sum_{j=1}^{n} L_{am}L_{ml}L_{bj}L_{jl}, \\
(L'LL'L)_{ab} &= \sum_{l=1}^{n} (L'L)_{al}(L'L)_{lb} = \sum_{l=1}^{n}\sum_{m=1}^{n}\sum_{j=1}^{n} L_{ma}L_{ml}L_{jl}L_{jb}.
\end{aligned} \tag{A.6}
$$

Using the expression for $(L)^4_{ab}$ in Equation (A.6),

$$\operatorname{tr}(L^4) = \sum_{i=1}^{n}\sum_{l=1}^{n}\sum_{m=1}^{n}\sum_{j=1}^{n} L_{im}L_{ml}L_{lj}L_{ji} = \sum_{i,j,l,m} P^{\gamma_n}_{ji}\mathbf{1}_{j>i}P^{\gamma_n}_{im}\mathbf{1}_{i>m}P^{\gamma_n}_{ml}\mathbf{1}_{m>l}P^{\gamma_n}_{lj}\mathbf{1}_{l>j} \\ = 0, \tag{A.7}$$

since there is no combination of indices i, j, l, m that jointly satisfy each of the indicator functions, as this would require $m < i < j < l < m$.

Using the expression for $(L^3L')_{ab}$ in Equation (A.6),

$$\begin{aligned}\operatorname{tr}(L^3L') &= \sum_{i=1}^{n}\sum_{l=1}^{n}\sum_{m=1}^{n}\sum_{j=1}^{n} L_{im}L_{mj}L_{jl}L_{il} = \sum_{i,j,l,m} P^{\gamma_n}_{im}\mathbf{1}_{i>m}P^{\gamma_n}_{mj}\mathbf{1}_{m>j}P^{\gamma_n}_{jl}\mathbf{1}_{j>l}P^{\gamma_n}_{il}\mathbf{1}_{i>l} \\ &= \sum_{l<j<m<i} P^{\gamma_n}_{im}P^{\gamma_n}_{mj}P^{\gamma_n}_{jl}P^{\gamma_n}_{il} = \sum_{i<j<l<m} P^{\gamma_n}_{ml}P^{\gamma_n}_{lj}P^{\gamma_n}_{ji}P^{\gamma_n}_{mi} \\ &= \sum_{i<j<l<m} P^{\gamma_n}_{ij}P^{\gamma_n}_{jl}P^{\gamma_n}_{im}P^{\gamma_n}_{lm}.\end{aligned} \tag{A.8}$$

The third equality follows since the product within the summations is non-zero only for $l < j < m < i$. The fourth equality follows by replacing the indices according to the dictionary $\{i : m, j : j, l : i, m : l\}$. The last equality follows from the symmetry of P^{γ_n}.

Using the expression for $(L^2L'^2)_{ab}$ in Equation (A.6),

$$
\begin{aligned}
\operatorname{tr}(L^2L'^2) &= \sum_{i=1}^{n}\sum_{l=1}^{n}\sum_{m=1}^{n}\sum_{j=1}^{n} L_{im}L_{ml}L_{ij}L_{jl} \\
&= \sum_{i,j,l,m} P_{im}^{\gamma_n}\mathbf{1}_{i>m}P_{ml}^{\gamma_n}\mathbf{1}_{m>l}P_{ij}^{\gamma_n}\mathbf{1}_{i>j}P_{jl}^{\gamma_n}\mathbf{1}_{j>l} \\
&= \sum_{i>j>l,i>m>l} P_{im}^{\gamma_n}P_{ml}^{\gamma_n}P_{ij}^{\gamma_n}P_{jl}^{\gamma_n} = \sum_{i>j>l,i>m>l} P_{ij}^{\gamma_n}P_{jl}^{\gamma_n}P_{lm}^{\gamma_n}P_{mi}^{\gamma_n} \\
&= \sum_{i>j=m>l} P_{ij}^{\gamma_n}P_{jl}^{\gamma_n}P_{lm}^{\gamma_n}P_{mi}^{\gamma_n} + \sum_{i>j>m>l} P_{ij}^{\gamma_n}P_{jl}^{\gamma_n}P_{lm}^{\gamma_n}P_{mi}^{\gamma_n} \\
&\quad + \sum_{i>m>j>l} P_{ij}^{\gamma_n}P_{jl}^{\gamma_n}P_{lm}^{\gamma_n}P_{mi}^{\gamma_n} \\
&= \sum_{i>j>l} P_{ij}^{\gamma_n}P_{jl}^{\gamma_n}P_{lj}^{\gamma_n}P_{ji}^{\gamma_n} + \sum_{i<j<l<m} P_{ml}^{\gamma_n}P_{li}^{\gamma_n}P_{ij}^{\gamma_n}P_{jm}^{\gamma_n} \\
&\quad + \sum_{i>m>j>l} P_{ij}^{\gamma_n}P_{jl}^{\gamma_n}P_{lm}^{\gamma_n}P_{mi}^{\gamma_n} \\
&= \sum_{i>j>l} P_{ij}^{\gamma_n}P_{jl}^{\gamma_n}P_{lj}^{\gamma_n}P_{ji}^{\gamma_n} + \sum_{i<j<l<m} P_{ml}^{\gamma_n}P_{li}^{\gamma_n}P_{ij}^{\gamma_n}P_{jm}^{\gamma_n} \\
&\quad + \sum_{i<j<l<m} P_{mj}^{\gamma_n}P_{ji}^{\gamma_n}P_{il}^{\gamma_n}P_{lm}^{\gamma_n} \\
&= \sum_{i<j<l} (P_{ij}^{\gamma_n})^2(P_{jl}^{\gamma_n})^2 + 2\sum_{i<j<l<m} P_{ij}^{\gamma_n}P_{il}^{\gamma_n}P_{jm}^{\gamma_n}P_{lm}^{\gamma_n}.
\end{aligned}
\tag{A.9}
$$

The third equality follows since the product within the summations is non-zero only when both $i > j > l$ and $i > m > l$. The fourth equality follows from the symmetry of P^{γ_n}. The sixth equality follows by replacing the indices in the second summation according to the dictionary $\{i : m, j : l, l : i, m : j\}$. The seventh equality follows by replacing the indices in the third summation according to the dictionary $\{i : m, j : j, l : i, m : l\}$. The last equality follows from the symmetry of P^{γ_n}.

Using the expression for $(L'LL'L)_{ab}$ in Equation (A.6),

$$
\begin{aligned}
\operatorname{tr}(L'LL'L) =& \sum_{i=1}^{n}\sum_{l=1}^{n}\sum_{m=1}^{n}\sum_{j=1}^{n} L_{mi}L_{ml}L_{jl}L_{ji} \\
=& \sum_{i,j,l,m} P_{mi}^{\gamma_n}\mathbf{1}_{m>i}P_{ml}^{\gamma_n}\mathbf{1}_{m>l}P_{jl}^{\gamma_n}\mathbf{1}_{j>l}P_{ji}^{\gamma_n}\mathbf{1}_{j>i} \\
=& \sum_{i,j,l,m} P_{ij}^{\gamma_n}\mathbf{1}_{i>j}P_{l>j}^{\gamma_n}\mathbf{1}_{l>j}P_{lm}^{\gamma_n}\mathbf{1}_{l>m}P_{im}^{\gamma_n}\mathbf{1}_{i>m} \\
=& \sum_{m=j<i=l} P_{ij}^{\gamma_n}P_{lj}^{\gamma_n}P_{lm}^{\gamma_n}P_{im}^{\gamma_n} + \sum_{m=j<l<i} P_{ij}^{\gamma_n}P_{lj}^{\gamma_n}P_{lm}^{\gamma_n}P_{im}^{\gamma_n} \\
&+ \sum_{m=j<i<l} P_{ij}^{\gamma_n}P_{lj}^{\gamma_n}P_{lm}^{\gamma_n}P_{im}^{\gamma_n} + \sum_{j<m<i=l} P_{ij}^{\gamma_n}P_{lj}^{\gamma_n}P_{lm}^{\gamma_n}P_{im}^{\gamma_n} \\
&+ \sum_{m<j<i=l} P_{ij}^{\gamma_n}P_{lj}^{\gamma_n}P_{lm}^{\gamma_n}P_{im}^{\gamma_n} + \sum_{m<j<l<i} P_{ij}^{\gamma_n}P_{lj}^{\gamma_n}P_{lm}^{\gamma_n}P_{im}^{\gamma_n} \\
&+ \sum_{m<j<i<l} P_{ij}^{\gamma_n}P_{lj}^{\gamma_n}P_{lm}^{\gamma_n}P_{im}^{\gamma_n} + \sum_{j<m<l<i} P_{ij}^{\gamma_n}P_{lj}^{\gamma_n}P_{lm}^{\gamma_n}P_{im}^{\gamma_n} \\
&+ \sum_{j<m<i<l} P_{ij}^{\gamma_n}P_{lj}^{\gamma_n}P_{lm}^{\gamma_n}P_{im}^{\gamma_n} \qquad \text{(A.10)} \\
=& \sum_{j<i}(P_{ij}^{\gamma_n})^4 + \sum_{j<l<i}(P_{ij}^{\gamma_n})^2(P_{lj}^{\gamma_n})^2 + \sum_{j<i<l}(P_{ij}^{\gamma_n})^2(P_{lj}^{\gamma_n})^2 \\
&+ \sum_{j<m<i}(P_{ij}^{\gamma_n})^2(P_{im}^{\gamma_n})^2 + \sum_{m<j<i}(P_{ij}^{\gamma_n})^2(P_{im}^{\gamma_n})^2 \\
&+ 4\sum_{i<j<l<m} P_{li}^{\gamma_n}P_{mi}^{\gamma_n}P_{lj}^{\gamma_n}P_{mj}^{\gamma_n} \\
=& \sum_{j<i}(P_{ij}^{\gamma_n})^4 + 2\sum_{i<j<l}(P_{li}^{\gamma_n})^2(P_{ji}^{\gamma_n})^2 \\
&+ \sum_{i<j<l}(P_{li}^{\gamma_n})^2(P_{lj}^{\gamma_n})^2 + 4\sum_{i<j<l<m} P_{li}^{\gamma_n}P_{mi}^{\gamma_n}P_{lj}^{\gamma_n}P_{mj}^{\gamma_n} \\
=& \sum_{i<j}(P_{ij}^{\gamma_n})^4 + 2\sum_{i<j<l}\Big((P_{ij}^{\gamma_n})^2(P_{il}^{\gamma_n})^2 + (P_{il}^{\gamma_n})^2(P_{jl}^{\gamma_n})^2\Big) \\
&+ 4\sum_{i<j<l<m} P_{il}^{\gamma_n}P_{jl}^{\gamma_n}P_{im}^{\gamma_n}P_{jm}^{\gamma_n}.
\end{aligned}
$$

The third equality follows by replacing the indices in the summation according to the dictionary $\{i : j, j : i, l : m, m : l\}$. The fourth equality follows since the product within

the summation is non-zero only when both $i > j, m$ and $l > j, m$. The fifth equality follows from replacing the indices in the last four summations in the previous line according to the dictionaries $\{i : m, j : j, l : l, m : i\}$, $\{i : l, j : j, l : m, m : i\}$, $\{i : m, j : i, l : l, m : j\}$, $\{i : l, j : i, l : m, m : j\}$, respectively. The sixth equality follows from replacing the indices in the second, third, fourth and fifth summations in the previous line according to the dictionaries $\{i : l, j : i, l : j\}$, $\{i : j, j : i, l : l\}$, $\{i : l, j : i, m : j\}$, $\{i : l, j : j, m : i\}$, respectively. The last equality follows by the symmetry of P^{γ_n}.

Let $\mathfrak{S} := \sum_{i<j<l<m} P_{il}^{\gamma_n} P_{jl}^{\gamma_n} P_{im}^{\gamma_n} P_{jm}^{\gamma_n} + P_{ij}^{\gamma_n} P_{jl}^{\gamma_n} P_{im}^{\gamma_n} P_{lm}^{\gamma_n} + P_{ij}^{\gamma_n} P_{il}^{\gamma_n} P_{jm}^{\gamma_n} P_{lm}^{\gamma_n}$. Substituting the expressions in Equations (A.7)–(A.10) into Equation (A.5) yields

$$\begin{aligned}
\operatorname{tr}((P^{\gamma_n} - G)^4) =&8 \sum_{i<j<l<m} P_{ij}^{\gamma_n} P_{jl}^{\gamma_n} P_{im}^{\gamma_n} P_{lm}^{\gamma_n} \\
&+ 4 \left(\sum_{i<j<l} (P_{ij}^{\gamma_n})^2 (P_{jl}^{\gamma_n})^2 + 2 \sum_{i<j<l<m} P_{ij}^{\gamma_n} P_{il}^{\gamma_n} P_{jm}^{\gamma_n} P_{lm}^{\gamma_n} \right) \\
&+ 2 \left(\sum_{i<j} (P_{ij}^{\gamma_n})^4 + 2 \sum_{i<j<l} \left((P_{ij}^{\gamma_n})^2 (P_{il}^{\gamma_n})^2 + (P_{il}^{\gamma_n})^2 (P_{jl}^{\gamma_n})^2 \right) \right. \\
&\qquad \left. + 4 \sum_{i<j<l<m} P_{il}^{\gamma_n} P_{jl}^{\gamma_n} P_{im}^{\gamma_n} P_{jm}^{\gamma_n} \right) \\
=&2 \sum_{i<j} (P_{ij}^{\gamma_n})^4 \\
&+ 4 \sum_{i<j<l} \left((P_{ij}^{\gamma_n})^2 (P_{jl}^{\gamma_n})^2 + (P_{il}^{\gamma_n})^2 (P_{jl}^{\gamma_n})^2 + (P_{ij}^{\gamma_n})^2 (P_{il}^{\gamma_n})^2 \right) \\
&+ 8\mathfrak{S}.
\end{aligned}$$

Next, by the triangle inequality and Lemma 1 (vi),

$$\begin{aligned}
|\mathfrak{S}| \leq& \frac{1}{4} \sum_{i<j} (P_{ij}^{\gamma_n})^4 \\
&+ \frac{1}{2} \sum_{i<j<l} \left((P_{ij}^{\gamma_n})^2 (P_{jl}^{\gamma_n})^2 + (P_{il}^{\gamma_n})^2 (P_{jl}^{\gamma_n})^2 + (P_{ij}^{\gamma_n})^2 (P_{il}^{\gamma_n})^2 \right) \\
&+ \frac{1}{8} \operatorname{tr}((P^{\gamma_n} - G)^4) \\
\leq& C r_n.
\end{aligned} \tag{A.11}$$

Take $\{u_i\}$ to be a sequence of i.i.d. mean-zero and unit variance random variables. Define

$$\Delta_1 := \sum_{i<j<l} \left(P_{ij}^{\gamma_n} P_{il}^{\gamma_n} u_j u_l + P_{ij}^{\gamma_n} P_{jl}^{\gamma_n} u_i u_l + P_{il}^{\gamma_n} P_{jl}^{\gamma_n} u_i u_j \right),$$

$$\Delta_2 := \sum_{i<j<l} \left(P_{ij}^{\gamma_n} P_{il}^{\gamma_n} u_j u_l + P_{ij}^{\gamma_n} P_{jl}^{\gamma_n} u_i u_l \right), \Delta_3 := \sum_{i<j<l} \left(P_{il}^{\gamma_n} P_{jl}^{\gamma_n} u_i u_j \right).$$

Then by Lemma 1 (vi),

$$\begin{aligned}
\mathbb{E}[\Delta_3^2] &= \sum_{i<j<l} (P_{il}^{\gamma_n})^2 (P_{jl}^{\gamma_n})^2 + 2 \sum_{i<j<l<m} P_{il}^{\gamma_n} P_{jl}^{\gamma_n} P_{im}^{\gamma_n} P_{jm}^{\gamma_n} \\
&\leq r_n + 2 \sum_{i<j<l<m} P_{il}^{\gamma_n} P_{jl}^{\gamma_n} P_{im}^{\gamma_n} P_{jm}^{\gamma_n}.
\end{aligned}$$

Furthermore,

$$\mathbb{E}[\Delta_2 \Delta_3] = \sum_{i<j<l<m} P_{ij}^{\gamma_n} P_{il}^{\gamma_n} P_{jm}^{\gamma_n} P_{lm}^{\gamma_n} + \sum_{i<j<l<m} P_{ij}^{\gamma_n} P_{jl}^{\gamma_n} P_{im}^{\gamma_n} P_{lm}^{\gamma_n},$$

and

$$\begin{aligned}
\mathbb{E}[\Delta_2^2] &= \sum_{\{i,l\}<j<k} P_{ij}^{\gamma_n} P_{il}^{\gamma_n} P_{mj}^{\gamma_n} P_{ml}^{\gamma_n} + \sum_{i<\{j,l\}<m} P_{ij}^{\gamma_n} P_{jm}^{\gamma_n} P_{il}^{\gamma_n} P_{lm}^{\gamma_n} \\
&\quad + \sum_{i<j<l<m} P_{ij}^{\gamma_n} P_{im}^{\gamma_n} P_{jl}^{\gamma_n} P_{lm}^{\gamma_n} + \sum_{l<i<j<m} P_{ij}^{\gamma_n} P_{jm}^{\gamma_n} P_{li}^{\gamma_n} P_{lm}^{\gamma_n} \\
&= \sum_{i<j<l} (P_{ij}^{\gamma_n})^2 (P_{il}^{\gamma_n})^2 + \sum_{i<j<l} (P_{ij}^{\gamma_n})^2 (P_{jl}^{\gamma_n})^2 + 2 \sum_{i<m<j<l} P_{ij}^{\gamma_n} P_{il}^{\gamma_n} P_{mj}^{\gamma_n} P_{ml}^{\gamma_n} \\
&\quad + 2 \sum_{i<j<l<m} P_{ij}^{\gamma_n} P_{jm}^{\gamma_n} P_{il}^{\gamma_n} P_{lm}^{\gamma_n} \\
&\quad + \sum_{i<j<l<m} P_{ij}^{\gamma_n} P_{im}^{\gamma_n} P_{jl}^{\gamma_n} P_{lm}^{\gamma_n} + \sum_{i<j<l<m} P_{jl}^{\gamma_n} P_{lm}^{\gamma_n} P_{ij}^{\gamma_n} P_{im}^{\gamma_n} \\
&= \sum_{i<j<l} (P_{ij}^{\gamma_n})^2 (P_{il}^{\gamma_n})^2 + \sum_{i<j<l} (P_{ij}^{\gamma_n})^2 (P_{jl}^{\gamma_n})^2 + 2\mathfrak{S} \\
&\leq C r_n,
\end{aligned}$$

where the last inequality follows from Lemma 1 (vi) and Equation (A.11). Since $\Delta_1 = \Delta_2 + \Delta_3$, $\mathbb{E}[\Delta_1^2] = \mathbb{E}[\Delta_2^2] + \mathbb{E}[\Delta_3^2] + 2\mathbb{E}[\Delta_2 \Delta_3] \leq C r_n + 2\mathfrak{S} \leq C r_n$. Hence by the triangle

inequality and the expression for $\mathbb{E}[\Delta_3^2]$,

$$\left|\sum_{i<j<l<m} P_{il}^{\gamma_n} P_{jl}^{\gamma_n} P_{im}^{\gamma_n} P_{jm}^{\gamma_n}\right| \leq C\left(\mathbb{E}[\Delta_3^2] + r_n\right) \leq C\left(\mathbb{E}[(\Delta_1 - \Delta_2)^2] + r_n\right)$$
$$\leq C(\mathbb{E}[\Delta_1^2] + \mathbb{E}[\Delta_2^2] + r_n)$$
$$\leq C r_n.$$

□

Lemma 3. Let $\gamma_- > 0$ and $\Gamma_n = \Gamma(\gamma_-) := \{\gamma_n \in \Re : \gamma_n \geq 0 \text{ if } r_n = k_n, \text{ and } \gamma_n \geq \gamma_- > 0 \text{ if } r_n < k_n\}$. Then if P^{γ_n} is a non-diagonal matrix,[1]

$$\gamma_n^* := \max \arg\max_{\gamma_n \in \Gamma_n} \sum_{i=1}^n \sum_{j \neq i} (P_{ij}^{\gamma_n})^2 \in [0, \infty)$$

exists.

Proof. Notice that

$$\begin{aligned}
\sum_{i=1}^n \sum_{j \neq i} (P_{ij}^{\gamma_n})^2 &= \sum_{i=1}^n \sum_{j=1}^n (P_{ij}^{\gamma_n})^2 - \sum_{i=1}^n (P_{ii}^{\gamma_n})^2 \\
&= \sum_{i=1}^n (P^{\gamma_n})_{ii}^2 - \sum_{i=1}^n (P_{ii}^{\gamma_n})^2 \\
&= \sum_{l=1}^{r_n} \left(\frac{D_{ll}^2}{D_{ll}^2 + \gamma_n}\right)^2 - \sum_{i=1}^n \left(\sum_{l=1}^{r_n} \frac{D_{ll}^2}{D_{ll}^2 + \gamma_n} U_{il}^2\right)^2,
\end{aligned}$$

where the third equality follows from Lemma 1 (iv) and Equation (A.3). Since

$$\lim_{\gamma_n \to \infty} \frac{D_{ll}^2}{D_{ll}^2 + \gamma_n} = 0,$$

and $U_{il}^2 \leq 1$ for $l = 1, \ldots, r_n$ and $i = 1, \ldots, n$, it follows that

$$\lim_{\gamma_n \to \infty} \sum_{i=1}^n \sum_{j \neq i} (P_{ij}^{\gamma_n})^2 = 0.$$

[1]We note that Assumption 3 excludes the case where P^{γ_n} is diagonal, so that γ_n^* exists under the assumptions made in this paper.

Hence, the maximum is not attained for arbitrarily large γ_n. Since P^{γ_n} is a non-diagonal matrix by assumption, $\sum_{i=1}^{n}\sum_{j\neq i}(P_{ij}^{\gamma_n})^2$ is strictly positive. This leaves a compact set over which the non-zero $\sum_{i=1}^{n}\sum_{j\neq i}(P_{ij}^{\gamma_n})^2$ is maximised, such that a maximiser exists.

□

Lemma 4. Under Assumptions 1, 2 and the null hypothesis in Equation (2), $|\hat{\Phi}_{\gamma_n} - \Phi_{\gamma_n}| \xrightarrow{p} 0$.

Proof. Defining $\eta_i := \varepsilon_i^2 - \mathbb{E}[\varepsilon_i^2]$, one can write

$$\hat{\Phi}_{\gamma_n} - \Phi_{\gamma_n} = \frac{2}{r_n}\sum_{i=1}^{n}\sum_{j\neq i}(P_{ij}^{\gamma_n})^2\left(\eta_i\eta_j + \mathbb{E}[\varepsilon_j^2]\eta_i + \mathbb{E}[\varepsilon_i^2]\eta_j\right),$$

and it follows that

$$\begin{aligned}\left|\hat{\Phi}_{\gamma_n} - \Phi_{\gamma_n}\right| \leq & \frac{2}{r_n}\left|\sum_{i=1}^{n}\sum_{j\neq i}(P_{ij}^{\gamma_n})^2\eta_i\eta_j\right| + \frac{2}{r_n}\left|\sum_{i=1}^{n}\sum_{j\neq i}(P_{ij}^{\gamma_n})^2\mathbb{E}[\varepsilon_j^2]\eta_i\right| \\ & + \frac{2}{r_n}\left|\sum_{i=1}^{n}\sum_{j\neq i}^{n}(P_{ij}^{\gamma_n})^2\mathbb{E}[\varepsilon_i^2]\eta_j\right| \\ \equiv & A_1 + A_2 + A_3.\end{aligned} \tag{A.12}$$

Consider each of $\mathbb{E}[A_1^2]$, $\mathbb{E}[A_2^2]$, and $\mathbb{E}[A_3^2]$ in turn.

$$\begin{aligned}\mathbb{E}[A_1^2] =& \frac{4}{r_n^2}\sum_{i=1}^{n}\sum_{j\neq i}\sum_{h=1}^{n}\sum_{g\neq h}(P_{ij}^{\gamma_n})^2(P_{hg}^{\gamma_n})^2\mathbb{E}[\eta_i\eta_j\eta_h\eta_g] \\ =& \frac{4}{r_n^2}\sum_{i=1}^{n}\sum_{j\neq i}\sum_{h\neq i}\sum_{g\neq h}(P_{ij}^{\gamma_n})^2(P_{hg}^{\gamma_n})^2\mathbb{E}[\eta_i\eta_j\eta_h\eta_g] \\ & + \frac{4}{r_n^2}\sum_{i=1}^{n}\sum_{j\neq i}\sum_{g\neq i}(P_{ij}^{\gamma_n})^2(P_{ig}^{\gamma_n})^2\mathbb{E}[\eta_i^2\eta_j\eta_g] \\ =& \frac{4}{r_n^2}\sum_{i=1}^{n}\sum_{j\neq i}\sum_{h\neq i}\sum_{g\notin\{h,i\}}(P_{ij}^{\gamma_n})^2(P_{hg}^{\gamma_n})^2\mathbb{E}[\eta_i]\mathbb{E}[\eta_j\eta_h\eta_g] \\ & + \frac{4}{r_n^2}\sum_{i=1}^{n}\sum_{j\neq i}\sum_{h\neq i}(P_{ij}^{\gamma_n})^2(P_{ih}^{\gamma_n})^2\mathbb{E}[\eta_i^2\eta_j\eta_h] \\ & + \frac{4}{r_n^2}\sum_{i=1}^{n}\sum_{j\neq i}\sum_{g\neq i}(P_{ij}^{\gamma_n})^2(P_{ig}^{\gamma_n})^2\mathbb{E}[\eta_i^2\eta_j\eta_g]\end{aligned}$$

$$
\begin{aligned}
&= \frac{8}{r_n^2} \sum_{i=1}^{n} \sum_{j \neq i} \sum_{h \neq i} (P_{ij}^{\gamma_n})^2 (P_{ih}^{\gamma_n})^2 \mathbb{E}[\eta_i^2 \eta_j \eta_h] \\
&= \frac{8}{r_n^2} \sum_{i=1}^{n} \sum_{j \neq i} \sum_{h \notin \{i,j\}} (P_{ij}^{\gamma_n})^2 (P_{ih}^{\gamma_n})^2 \mathbb{E}[\eta_i^2] \mathbb{E}[\eta_j] \mathbb{E}[\eta_h] \\
&\quad + \frac{8}{r_n^2} \sum_{i=1}^{n} \sum_{j \neq i} (P_{ij}^{\gamma_n})^4 \mathbb{E}[\eta_i^2] \mathbb{E}[\eta_j^2] \\
&= \frac{8}{r_n^2} \sum_{i=1}^{n} \sum_{j \neq i} (P_{ij}^{\gamma_n})^4 \mathbb{E}[\eta_i^2] \mathbb{E}[\eta_j^2] \\
&\leq \frac{C}{r_n^2} \sum_{i=1}^{n} \sum_{j \neq i} (P_{ij}^{\gamma_n})^4 \\
&\leq \frac{C}{r_n},
\end{aligned}
$$

The fourth equality follows from $\mathbb{E}[\eta_i] = 0$ and the symmetry of P^{γ_n}. The sixth equality follows from $\mathbb{E}[\eta_j] = 0$. The first inequality follows from Assumption 1, which implies $\sup_{i \in \mathbb{N}} \mathbb{E}[\eta_i^2] < \infty$. The second inequality follows from Lemma 1 (vi). Next, concerning $\mathbb{E}[A_2^2]$ one has

$$
\begin{aligned}
\mathbb{E}[A_2^2] &= \frac{4}{r_n^2} \sum_{i=1}^{n} \sum_{j \neq i} \sum_{h=1}^{n} \sum_{g \neq h} (P_{ij}^{\gamma_n})^2 (P_{hg}^{\gamma_n})^2 \mathbb{E}[\varepsilon_j^2] \mathbb{E}[\varepsilon_g^2] \mathbb{E}[\eta_i \eta_h] \\
&= \frac{4}{r_n^2} \sum_{i=1}^{n} \sum_{j \neq i} \sum_{h \neq i} \sum_{g \neq h} (P_{ij}^{\gamma_n})^2 (P_{hg}^{\gamma_n})^2 \mathbb{E}[\varepsilon_j^2] \mathbb{E}[\varepsilon_g^2] \mathbb{E}[\eta_i] \mathbb{E}[\eta_h] \\
&+ \frac{4}{r_n^2} \sum_{i=1}^{n} \sum_{j \neq i} \sum_{g \neq i} (P_{ij}^{\gamma_n})^2 (P_{ig}^{\gamma_n})^2 \mathbb{E}[\varepsilon_j^2] \mathbb{E}[\varepsilon_g^2] \mathbb{E}[\eta_i^2] \\
&= \frac{4}{r_n^2} \sum_{i=1}^{n} \sum_{j \neq i} \sum_{g \neq i} (P_{ij}^{\gamma_n})^2 (P_{ig}^{\gamma_n})^2 \mathbb{E}[\varepsilon_j^2] \mathbb{E}[\varepsilon_g^2] \mathbb{E}[\eta_i^2] \\
&\leq \frac{C}{r_n^2} \sum_{i=1}^{n} \sum_{j \neq i} \sum_{g \neq i} (P_{ij}^{\gamma_n})^2 (P_{ig}^{\gamma_n})^2 \\
&\leq \frac{C}{r_n},
\end{aligned}
$$

by the same reasoning that led to the penultimate display. Finally, similar arguments imply that $\mathbb{E}[A_3^2] \leq \frac{C}{r_n}$ such that by Markov's inequality and Equation (A.12) it follows that $\hat{\Phi}_{\gamma_n} - \Phi_{\gamma_n} = O_p(r_n^{-1/2})$. Thus, by Assumption 2, $|\hat{\Phi}_{\gamma_n} - \Phi_{\gamma_n}| \xrightarrow{p} 0$. □

A.2 Proof of Proposition 1

As observed after the statement of Proposition 1, it suffices to prove its part 2. Thus, assume that there exists a $\delta' \in (0,1)$ such that $\frac{1}{k_n}|\mathcal{A}_n(\delta')| \to 0$. Notice that the sum in Assumption 3 with $\gamma_n = 0$ can be written as

$$\begin{aligned}
\frac{1}{k_n}\sum_{i=1}^{n}\sum_{j\neq i} P_{ij}^2 &= \frac{1}{k_n}\left(\sum_{i=1}^{n} P_{ii} - \sum_{i=1}^{n} P_{ii}^2\right) \\
&= \frac{1}{k_n}\left(k_n - \sum_{i\in\mathcal{A}_n^c(\delta')} P_{ii}^2 - \sum_{i\in\mathcal{A}_n(\delta')} P_{ii}^2\right) \\
&\geq \frac{1}{k_n}\left(k_n - (1-\delta')\sum_{i\in\mathcal{A}_n^c(\delta')} P_{ii} - \sum_{i\in\mathcal{A}_n(\delta')} P_{ii}^2\right) \\
&\geq \frac{1}{k_n}\left(k_n - (1-\delta')k_n - |\mathcal{A}_n(\delta')|\right) \\
&\geq \frac{1}{k_n}\left(k_n\delta' - |\mathcal{A}_n(\delta')|\right),
\end{aligned}$$

where the first equality uses the idempotency and symmetry of P, and the second equality uses the fact that the trace of P is equal to its rank k_n. Thus, if $\frac{1}{k_n}|\mathcal{A}_n(\delta')| \to 0$,

$$\liminf_{n\to\infty} \frac{1}{k_n}\sum_{i=1}^{n}\sum_{j\neq i}(P_{ij}^{\gamma_n^*})^2 \geq \liminf_{n\to\infty}\frac{1}{k_n}\sum_{i=1}^{n}\sum_{j\neq i} P_{ij}^2 \geq \delta' > 0,$$

and hence Assumption 3 is satisfied.

□

A.3 Proof of Proposition 2

The following preparatory lemma is likely well-known but we state it here for ease of reference. Its proof is a simple consequence of the Marchenko-Pastur theorem.[2] Denote by $\lambda_{n,1} \geq \ldots \geq \lambda_{n,n} \geq 0$ the eigenvalues of $\frac{ZZ'}{k_n}$, where $\lambda_{n,i} = \frac{D_{ii}^2}{k_n}$ in our earlier notation on p. 3, and by F_n the empirical distribution of these.

[2]We thank Alexei Onatski for suggesting this.

Lemma 5. Let Z be an $n \times k_n$ matrix with i.i.d. entries of mean zero and variance one. In addition, let $p > 2$ satisfy $\mathbb{E}|Z_{11}|^{4p} < \infty$ and let $k_n = \tau n$ for $\tau \in [1, \infty)$. Then

1. $\frac{1}{n}\sum_{i=1}^{n} \lambda_{n,i} \to 1$ almost surely.
2. $\frac{1}{n}\text{tr}\left[\left(\frac{ZZ'}{n}\right)^2\right] = \frac{\tau^2}{n}\text{tr}\left[\left(\frac{ZZ'}{k_n}\right)^2\right] = \frac{\tau^2}{n}\sum_{i=1}^{n} \lambda_{n,i}^2 \to \tau^2 + \tau$ almost surely.
3. $\frac{1}{n}\sum_{i=1}^{n}\left[\left(\frac{ZZ'}{n}\right)_{ii}\right]^2 = \frac{\tau^2}{n}\sum_{i=1}^{n}\left[\left(\frac{ZZ'}{k_n}\right)_{ii}\right]^2 \to \tau^2$ almost surely.

Proof. Denote by F_τ the Marchenko-Pastur distribution with "dimension-to-sample-ratio" $1/\tau$ (observe that the dimension of ZZ' is $n \times n$ whereas each entry is based on a "sample size" of k_n). By the Marchenko-Pastur theorem (as stated in Theorem 3.6 of Bai and Silverstein (2010)), F_n almost surely converges weakly to F_τ. In addition, by the Bai-Yin-Krishnaiah law (as stated in Theorem 5.8 of Bai and Silverstein (2010)) it follows that $\lambda_{n,1} \to (1+\tau^{-1/2})^2$ almost surely. Hence, outside a set of probability zero for n sufficiently large F_n has compact support and therefore $F_n \circ (x \mapsto x^a)^{-1}$ is uniformly integrable for all $a \in (0, \infty)$.

We first establish part 1. This follows by the observations just made because

$$\frac{1}{n}\sum_{i=1}^{n} \lambda_{n,i} = \int x dF_n \to \int x dF_\tau = 1 \qquad \text{almost surely,}$$

where the last equality follows from, e.g., Lemma 3.1 in Bai and Silverstein (2010).

To prove part 2., note that since $k_n/n = \tau$

$$\frac{1}{n}\text{tr}\left[\left(\frac{ZZ'}{n}\right)^2\right] = \frac{\tau^2}{n}\text{tr}\left[\left(\frac{ZZ'}{k_n}\right)^2\right] = \frac{\tau^2}{n}\sum_{i=1}^{n}\lambda_{n,i}^2.$$

The almost sure convergence to $\tau^2 + \tau$ follows from the observations prior to the previous display, i.e.

$$\frac{\tau^2}{n}\sum_{i=1}^{n}\lambda_{n,i}^2 = \tau^2\int x^2 dF_n \to \tau^2 \int x^2 dF_\tau = \tau^2(1+1/\tau) = \tau^2 + \tau \qquad \text{almost surely,}$$

where the second-to-last equality follows from, e.g., Lemma 3.1 in Bai and Silverstein (2010).

Next, to establish part 3., note that

$$\mathbb{E}\left[\left(\frac{ZZ'}{k_n}\right)_{ii}\right]^2 = \frac{1}{k_n^2}\sum_{j=1}^{k_n}\mathbb{E}Z_{ij}^4 + \frac{1}{k_n^2}\sum_{j=1}^{k_n}\sum_{l\neq j}^{k_n}\mathbb{E}Z_{ij}^2\mathbb{E}Z_{il}^2 \to 1 \qquad \text{for } i=1,\dots,n$$

and that the $\left[\left(\frac{ZZ'}{k_n}\right)_{ii}\right]^2$ are independent across $i=1,\dots,n$. Therefore, an application of Markov's inequality along with the Marcinkiewicz-Zygmund inequality yields that there exists a $c_p>0$ such that for every $\varepsilon>0$

$$\begin{aligned}
\mathbb{P}\left(\left|\frac{1}{n}\sum_{i=1}^{n}\left[\left(\frac{ZZ'}{k_n}\right)_{ii}\right]^2 - \mathbb{E}\left[\left(\frac{ZZ'}{k_n}\right)_{11}\right]^2\right| \geq \varepsilon\right) &\leq c_p\frac{\mathbb{E}\left(\sum_{i=1}^{n}\left(\left[\left(\frac{ZZ'}{k_n}\right)_{ii}\right]^2 - \mathbb{E}\left[\left(\frac{ZZ'}{k_n}\right)_{11}\right]^2\right)^2\right)^{p/2}}{\varepsilon^p n^p} \\
&= c_p\frac{n^{p/2}\mathbb{E}\left(\frac{1}{n}\sum_{i=1}^{n}\left(\left[\left(\frac{ZZ'}{k_n}\right)_{ii}\right]^2 - \mathbb{E}\left[\left(\frac{ZZ'}{k_n}\right)_{11}\right]^2\right)^2\right)^{p/2}}{\varepsilon^p n^p} \\
&\leq c_p\frac{n^{p/2}\mathbb{E}\left|\left[\left(\frac{ZZ'}{k_n}\right)_{11}\right]^2 - \mathbb{E}\left[\left(\frac{ZZ'}{k_n}\right)_{11}\right]^2\right|^p}{\varepsilon^p n^p},
\end{aligned}$$

where the second inequality follows by Jensen's inequality as well as the $\left[\left(\frac{ZZ'}{k_n}\right)_{ii}\right]^2$ being identically distributed. Another application of Jensen's inequality shows that the right-hand side is bounded from above by $c(p,\varepsilon)n^{-p/2}$ [here $c(p,\varepsilon)$ is a non-negative constant depending only on p and ε]. Therefore, part 3. follows from the Borel-Cantelli lemma. □

Proof of Proposition 2. Since the entries of Z have a distribution that is absoluteley continuous with respect to the Lebesgue measure, the rank of Z is n with probability one. Hence, we show that there exists a $\gamma_- > 0$ and a sequence γ_n satisfying $\gamma_n \geq \gamma_-$ for all $n \in \mathbb{N}$ such that

$$\liminf_{n\to\infty}\frac{1}{n}\sum_{i=1}^{n}\sum_{j\neq i}^{n}\left(P_{ij}^{\gamma_n}\right)^2 > 0 \qquad \text{almost surely.} \tag{A.13}$$

Observe that

$$\frac{1}{n}\sum_{i=1}^{n}\sum_{j\neq i}^{n}\left(P_{ij}^{\gamma_n}\right)^2 = \frac{1}{n}\sum_{i=1}^{n}\left(P^{\gamma_n}\right)_{ii}^2 - \frac{1}{n}\sum_{i=1}^{n}\left(P_{ii}^{\gamma_n}\right)^2 = \frac{1}{n}\mathrm{tr}\left[\left(P^{\gamma_n}\right)^2\right] - \frac{1}{n}\sum_{i=1}^{n}\left(P_{ii}^{\gamma_n}\right)^2.$$

Therefore, (A.13) will follow if we establish the existence of a sequence γ_n, bounded away from zero, such that almost surely

$$\liminf_{n\to\infty} \frac{1}{n}\mathrm{tr}\left[\left(P^{\gamma_n}\right)^2\right] > \limsup_{n\to\infty} \frac{1}{n}\sum_{i=1}^{n}\left(P_{ii}^{\gamma_n}\right)^2. \tag{A.14}$$

It follows by (A.2) and its ensuing discussion that

$$\frac{1}{\tau}P^{\gamma_n} = U\frac{1}{\tau}\tilde{D}U' = U\check{D}U', \tag{A.15}$$

with $\check{D} = \frac{1}{\tau}\tilde{D}$ such that with $\gamma_n = \eta_n n$ for some $\eta_n \geq 0$

$$\check{D}_{ii} = \frac{1}{\tau}\frac{\lambda_{n,i}}{\lambda_{n,i} + \gamma_n/k_n} = \frac{\lambda_{n,i}}{\tau\lambda_{n,i} + \eta_n} \qquad \text{for } i = 1, \ldots n. \tag{A.16}$$

We begin by lower bounding the left-hand side of (A.14).

$$\begin{aligned}
\frac{1}{n}\mathrm{tr}\left[\left(P^{\gamma_n}\right)^2\right] &\geq \frac{1}{n}\mathrm{tr}\left[\left(\frac{1}{\eta_n n}ZZ'\right)^2\right] - \left|\frac{1}{n}\mathrm{tr}\left[\left(\frac{1}{\eta_n n}ZZ'\right)^2\right] - \frac{1}{n}\mathrm{tr}\left[\left(P^{\gamma_n}\right)^2\right]\right| \\
&= \frac{\tau^2}{n}\mathrm{tr}\left[\left(\frac{1}{\eta_n k_n}ZZ'\right)^2\right] - \frac{\tau^2}{n}\left|\mathrm{tr}\left[\left(\frac{1}{\eta_n k_n}ZZ'\right)^2\right] - \mathrm{tr}\left[\left(\frac{1}{\tau}P^{\gamma_n}\right)^2\right]\right| \\
&= \frac{\tau^2}{n\eta_n^2}\sum_{i=1}^{n}\lambda_{n,i}^2 - \frac{\tau^2}{n}\left|\frac{1}{\eta_n^2}\sum_{i=1}^{n}\lambda_{n,i}^2 - \sum_{i=1}^{n}\frac{\lambda_{n,i}^2}{(\tau\lambda_{n,i}+\eta_n)^2}\right|
\end{aligned}$$

Furthermore,

$$\left|\frac{1}{\eta_n^2}\sum_{i=1}^{n}\lambda_{n,i}^2 - \sum_{i=1}^{n}\frac{\lambda_{n,i}^2}{(\tau\lambda_{n,i}+\eta_n)^2}\right| = \frac{1}{\eta_n^2}\sum_{i=1}^{n}\lambda_{n,i}^2\left[1 - \frac{\eta_n^2}{(\tau\lambda_{n,i}+\eta_n)^2}\right],$$

where by Theorem 5.8 of Bai and Silverstein (2010) for $i = 1, \ldots, n$

$$\lambda_{n,i} \leq \lambda_{n,1} \to (1+\tau^{-1/2})^2 \qquad \text{almost surely}$$

such that for any $\varepsilon > 0$ we can and do choose $\eta_n = \frac{2\tau(1+\tau^{-1/2})^2}{\varepsilon} =: \eta_\varepsilon$ not depending on n to ensure that $\lambda_{n,1} \leq \varepsilon\tau^{-1}\eta_n$ eventually. Thus, almost surely eventually

$$\left|\frac{1}{\eta_\varepsilon^2}\sum_{i=1}^{n}\lambda_{n,i}^2 - \sum_{i=1}^{n}\frac{\lambda_{n,i}^2}{(\tau\lambda_{n,i}+\eta_\varepsilon)^2}\right| \leq \frac{1}{\eta_\varepsilon^2}\sum_{i=1}^{n}\lambda_{n,i}^2\left[1 - \frac{1}{(1+\varepsilon)^2}\right].$$

and it follows by part 2. of Lemma 5 that with $\gamma_{n,\varepsilon} = n\eta_\varepsilon$

$$\liminf_{n\to\infty}\frac{1}{n}\mathrm{tr}\left[\left(P^{\gamma_{n,\varepsilon}}\right)^2\right] \geq \liminf_{n\to\infty}\frac{\tau^2}{(1+\varepsilon)^2 n\eta_\varepsilon^2}\sum_{i=1}^{n}\lambda_{n,i}^2 = \frac{\tau^2+\tau}{(1+\varepsilon)^2\eta_\varepsilon^2} \qquad \text{almost surely,} \tag{A.17}$$

We now bound the right-hand side of (A.14) from above. To this end, observe that for $i = 1, \ldots, n$

$$\frac{1}{n}\sum_{i=1}^{n}\left(P_{ii}^{\gamma_{n,\varepsilon}}\right)^2 \le \frac{1}{n}\sum_{i=1}^{n}\left[\left(\frac{ZZ'}{\eta_\varepsilon n}\right)_{ii}\right]^2 + \left|\frac{1}{n}\sum_{i=1}^{n}\left[\left(\frac{ZZ'}{\eta_\varepsilon n}\right)_{ii}\right]^2 - \frac{1}{n}\sum_{i=1}^{n}\left(P_{ii}^{\gamma_{n,\varepsilon}}\right)^2\right|$$
$$= \frac{\tau^2}{n}\sum_{i=1}^{n}\left[\left(\frac{ZZ'}{\eta_\varepsilon k_n}\right)_{ii}\right]^2 + \frac{\tau^2}{n}\left|\sum_{i=1}^{n}\left[\left(\frac{ZZ'}{\eta_\varepsilon k_n}\right)_{ii}\right]^2 - \sum_{i=1}^{n}\left(\frac{1}{\tau}P_{ii}^{\gamma_{n,\varepsilon}}\right)^2\right|$$

with

$$\left|\sum_{i=1}^{n}\left[\left(\frac{ZZ'}{\eta_\varepsilon k_n}\right)_{ii}\right]^2 - \sum_{i=1}^{n}\left(\frac{1}{\tau}P_{ii}^{\gamma_{n,\varepsilon}}\right)^2\right| \le \max_{1\le i\le n}\left[\left(\frac{ZZ'}{\eta_\varepsilon k_n}\right)_{ii} + \frac{1}{\tau}P_{ii}^{\gamma_{n,\varepsilon}}\right]\sum_{i=1}^{n}\left|\left(\frac{ZZ'}{\eta_\varepsilon k_n}\right)_{ii} - \frac{1}{\tau}P_{ii}^{\gamma_{n,\varepsilon}}\right|.$$

By (A.15) and (A.16)

$$\left(\frac{ZZ'}{\eta_\varepsilon k_n}\right) - \frac{1}{\tau}P^{\gamma_n} = U\left(\eta_\varepsilon^{-1}\mathrm{diag}(\lambda_{n,1}, \ldots, \lambda_{n,n}) - \check{D}\right)U',$$

and since

$$\left(\eta_\varepsilon^{-1}\mathrm{diag}(\lambda_{n,1}, \ldots, \lambda_{n,n}) - \check{D}\right)_{ii} = \frac{\lambda_{n,i}}{\eta_\varepsilon} - \frac{\lambda_{n,i}}{\tau\lambda_{n,i} + \eta_\varepsilon} = \frac{\lambda_{n,i}}{\eta_\varepsilon}\left(1 - \frac{\eta_\varepsilon}{\tau\lambda_{n,i} + \eta_\varepsilon}\right) \ge 0 \qquad \text{for } i = 1, \ldots n$$

it follows that all eigenvalues of $\left(\frac{ZZ'}{\eta_\varepsilon k_n}\right) - \frac{1}{\tau}P^{\gamma_{n,\varepsilon}}$ are non-negative such that it is positive semi-definite. Hence, all its diagonal elements are non-negative; that is $\frac{1}{\tau}P_{ii}^{\gamma_{n,\varepsilon}} \le \left(\frac{ZZ'}{\eta_\varepsilon k_n}\right)_{ii}$ for $i = 1, \ldots, n$. Since the diagonal elements of a symmetric matrix are bounded from above by the largest eigenvalue, it follows Theorem 5.8 of Bai and Silverstein (2010) that, almost surely,

$$\limsup_{n\to\infty}\max_{1\le i\le n}\left[\left(\frac{ZZ'}{\eta_\varepsilon k_n}\right)_{ii} + \frac{1}{\tau}P_{ii}^{\gamma_{n,\varepsilon}}\right] \le \limsup_{n\to\infty}\max_{1\le i\le n} 2\left(\frac{ZZ'}{\eta_\varepsilon k_n}\right)_{ii} \le \limsup_{n\to\infty}\frac{2\lambda_{n,1}}{\eta_\varepsilon} = \frac{2(1+\tau^{-1/2})^2}{\eta_\varepsilon}.$$

Next, recalling that $\frac{1}{\tau}P_{ii}^{\gamma_{n,\varepsilon}} \le \left(\frac{ZZ'}{\eta_\varepsilon k_n}\right)_{ii}$ for $i = 1, \ldots, n$, it follows by the penultimate display,

$$\sum_{i=1}^{n}\left|\left(\frac{ZZ'}{\eta_\varepsilon k_n}\right)_{ii} - \frac{1}{\tau}P_{ii}^{\gamma_{n,\varepsilon}}\right| = \sum_{i=1}^{n}\frac{\lambda_{n,i}}{\eta_\varepsilon}\left(1 - \frac{\eta_\varepsilon}{\tau\lambda_{n,i} + \eta_\varepsilon}\right) \le \sum_{i=1}^{n}\frac{\lambda_{n,i}}{\eta_\varepsilon}\left(1 - \frac{1}{1+\varepsilon}\right),$$

almost surely (for n sufficiently large) implying that

$$\limsup_{n\to\infty}\frac{1}{n}\sum_{i=1}^{n}\left|\left(\frac{ZZ'}{\eta_\varepsilon k_n}\right)_{ii} - \frac{1}{\tau}P_{ii}^{\gamma_{n,\varepsilon}}\right| \le \frac{1}{\eta_\varepsilon}\left(1 - \frac{1}{1+\varepsilon}\right) \qquad \text{almost surely}$$

by part 1. of Lemma 5. Thus, in total, and using part 3. of Lemma 5,

$$\limsup_{n\to\infty} \frac{1}{n} \sum_{i=1}^{n} \left(P_{ii}^{\gamma_{n,\varepsilon}}\right)^2 \leq \frac{\tau^2}{\eta_\varepsilon^2} \left[1 + 2(1+\tau^{-1/2})^2 \left(1 - \frac{1}{1+\varepsilon}\right)\right] \qquad \text{almost surely.} \quad \text{(A.18)}$$

For any $\delta > 0$ one can ensure that the right-hand side in the previous display does not exceed $\frac{\tau^2}{\eta_\varepsilon^2}(1+\delta)$ by choosing ε sufficiently close to zero. Thus, together (A.17) and (A.18) yield (A.14) (with $\gamma_{n,\varepsilon}$ being the sought after sequence γ_n). □

A.4 Proof of Theorem 1

We begin by showing that

$$RJAR_{\gamma_n}^* := \frac{1}{\sqrt{r_n}\sqrt{\Phi_{\gamma_n}}} \sum_{i=1}^{n} \sum_{j\neq i} P_{ij}^{\gamma_n} \varepsilon_i \varepsilon_j \qquad \text{(A.19)}$$

converges in distribution to a standard normal for all sequences of γ_n that satisfy Assumption 3.[3] To this end, define $\mathcal{U}_n := 2\sum_{i=2}^{n}\sum_{j=1}^{i-1} P_{ij}^{\gamma_n}\varepsilon_i\varepsilon_j$, $s_n^2 := \mathbb{E}[\mathcal{U}_n^2]$ and note that by the symmetry of P^{γ_n}, $RJAR_{\gamma_n}^*$ can be written as

$$RJAR_{\gamma_n}^* = s_n^{-1}\mathcal{U}_n.$$

We proceed by establishing that $s_n^{-1}\mathcal{U}_n \xrightarrow{d} \mathcal{N}[0,1]$ as $n \to \infty$. This, in turn, follows from Hall and Heyde (1980, Corollary 3.1)) upon verifying that i) for all $\epsilon > 0$

$$s_n^{-2} \sum_{i=2}^{n} \mathbb{E}[Y_{ni}^2 I(|Y_{ni}| > \epsilon s_n)] \to 0 \qquad \text{as } n \to \infty, \qquad \text{(A.20)}$$

where $Y_{ni} = 2\sum_{j=1}^{i-1} P_{ij}^{\gamma_n}\varepsilon_i\varepsilon_j$, and ii)

$$s_n^{-2}\mathcal{V}_n^2 \xrightarrow{p} 1 \qquad \text{as } n \to \infty, \qquad \text{(A.21)}$$

where $\mathcal{V}_n^2 = \sum_{i=2}^{n} \mathbb{E}[Y_{ni}^2|\varepsilon_1, \ldots, \varepsilon_{i-1}]$. Consider first the condition in Equation (A.20) and write

$$\mathbb{E}[Y_{ni}^2] = 4\sum_{j=1}^{i-1}\sum_{h=1}^{i-1} P_{ij}^{\gamma_n} P_{ih}^{\gamma_n} \mathbb{E}[\varepsilon_i^2 \varepsilon_j \varepsilon_h] = 4\sum_{j=1}^{i-1} (P_{ij}^{\gamma_n})^2 \mathbb{E}[\varepsilon_i^2]\mathbb{E}[\varepsilon_j^2],$$

[3] We do not directly invoke Lemma 2 in Hansen and Kozbur (2014) because it would require strengthening the assumptions used in this paper.

so that, upon using that $\mathcal{U}_n = \sum_{i=2}^n Y_{ni}$ and $\mathbb{E}[Y_{ni}Y_{nj}] = 0$ for $i \neq j$, one gets

$$s_n^2 = \sum_{i=2}^{n} \mathbb{E}[Y_{ni}^2] = 4 \sum_{i=2}^{n} \sum_{j=1}^{i-1} (P_{ij}^{\gamma_n})^2 \mathbb{E}[\varepsilon_i^2] \mathbb{E}[\varepsilon_j^2]. \tag{A.22}$$

Furthermore,

$$\begin{aligned}
\mathbb{E}[Y_{ni}^4] =& 16 \sum_{j=1}^{i-1} \sum_{h=1}^{i-1} \sum_{m=1}^{i-1} \sum_{l=1}^{i-1} P_{ij}^{\gamma_n} P_{ih}^{\gamma_n} P_{im}^{\gamma_n} P_{il}^{\gamma_n} \mathbb{E}[\varepsilon_i^4 \varepsilon_j \varepsilon_h \varepsilon_m \varepsilon_l] \\
=& 16 \sum_{j=1}^{i-1} (P_{ij}^{\gamma_n})^4 \mathbb{E}[\varepsilon_i^4] \mathbb{E}[\varepsilon_j^4] + 48 \sum_{j=1}^{i-1} \sum_{h \neq j} (P_{ij}^{\gamma_n})^2 (P_{ih}^{\gamma_n})^2 \mathbb{E}[\varepsilon_i^4] \mathbb{E}[\varepsilon_j^2] \mathbb{E}[\varepsilon_h^2],
\end{aligned}$$

so that

$$\begin{aligned}
\sum_{i=2}^{n} \mathbb{E}[Y_{ni}^4] =& 16 \sum_{i=2}^{n} \sum_{j=1}^{i-1} (P_{ij}^{\gamma_n})^4 \mathbb{E}[\varepsilon_i^4] \mathbb{E}[\varepsilon_j^4] \\
&+ 48 \sum_{i=2}^{n} \sum_{j=1}^{i-1} \sum_{h \neq j} (P_{ij}^{\gamma_n})^2 (P_{ih}^{\gamma_n})^2 \mathbb{E}[\varepsilon_i^4] \mathbb{E}[\varepsilon_j^2] \mathbb{E}[\varepsilon_h^2].
\end{aligned}$$

It follows that

$$\begin{aligned}
\frac{\sum_{i=2}^n \mathbb{E}[Y_{ni}^4]}{s_n^4} &\leq C \frac{\sum_{i=2}^n \sum_{j=1}^{i-1} (P_{ij}^{\gamma_n})^4 + \sum_{i=2}^n \sum_{j=1}^{i-1} \sum_{h \neq j} (P_{ij}^{\gamma_n})^2 (P_{ih}^{\gamma_n})^2}{\left(\sum_{i=1}^n \sum_{j \neq i} (P_{ij}^{\gamma_n})^2\right)^2} \\
&\leq C \frac{\sum_{i=1}^n \sum_{j=1}^n (P_{ij}^{\gamma_n})^2 + \sum_{i=1}^n \sum_{j=1}^n (P_{ij}^{\gamma_n})^2 \sum_{h=1}^n (P_{ih}^{\gamma_n})^2}{\left(\sum_{i=1}^n \sum_{j \neq i} (P_{ij}^{\gamma_n})^2\right)^2} \\
&= C \frac{\sum_{i=1}^n (P^{\gamma_n})_{ii}^2 + \sum_{i=1}^n \left((P^{\gamma_n})_{ii}^2\right)^2}{\left(\sum_{i=1}^n \sum_{j \neq i} (P_{ij}^{\gamma_n})^2\right)^2} \\
&\leq C \frac{\sum_{i=1}^n (P^{\gamma_n})_{ii}^2 + \sum_{i=1}^n (P^{\gamma_n})_{ii}^2}{\left(\sum_{i=1}^n \sum_{j \neq i} (P_{ij}^{\gamma_n})^2\right)^2} \\
&\leq C \frac{\sum_{i=1}^n (P^{\gamma_n})_{ii}^2}{r_n^2} \\
&\leq C r_n^{1-2} \\
&\to 0,
\end{aligned} \tag{A.23}$$

where the first inequality follows from Assumption 1. The second inequality follows from Lemma 1 (v). The third inequality follows from Lemma 1 (i). The fourth inequality follows from Equation (A.1). The fifth inequality follows from Lemma 1 (iii). The limit holds by

Assumption 2 and Assumption 3. The condition in Equation (A.20) follows from Equation (A.23).

In order to verify the convergence in Equation (A.21) it suffices to show that

$$s_n^{-4}\mathbb{E}\left[(\mathcal{V}_n^2 - s_n^2)^2\right] = \frac{\mathbb{E}[\mathcal{V}_n^4] + s_n^4 - 2s_n^2\mathbb{E}[\mathcal{V}_n^2]}{s_n^4} = \frac{\mathbb{E}[\mathcal{V}_n^4]}{s_n^4} + 1 - 2\frac{\mathbb{E}[\mathcal{V}_n^2]}{s_n^2} \to 0.$$

Since $\mathcal{V}_n^2 = \sum_{i=2}^n \mathbb{E}[Y_{ni}^2|\varepsilon_1, \ldots, \varepsilon_{i-1}]$ it follows that

$$\mathbb{E}[\mathcal{V}_n^2] = \sum_{i=2}^n \mathbb{E}[Y_{ni}^2] = s_n^2,$$

the last equality following from Equation (A.22). It remains to be verified that

$$\frac{\mathbb{E}[\mathcal{V}_n^4]}{s_n^4} \to 1. \tag{A.24}$$

To this end, observe that

$$\mathcal{V}_n^2 = \sum_{i=2}^n \mathbb{E}[Y_{ni}^2|\varepsilon_1, \ldots, \varepsilon_{i-1}] = 4\sum_{i=2}^n\sum_{j=1}^{i-1}\sum_{h=1}^{i-1} P_{ij}^{\gamma_n}P_{ih}^{\gamma_n}\mathbb{E}[\varepsilon_i^2]\varepsilon_j\varepsilon_h,$$

such that

$$\mathcal{V}_n^4 = 16\sum_{i=2}^n\sum_{j=2}^n\sum_{h=1}^{i-1}\sum_{l=1}^{i-1}\sum_{m=1}^{j-1}\sum_{w=1}^{j-1} P_{ih}^{\gamma_n}P_{il}^{\gamma_n}P_{jm}^{\gamma_n}P_{jw}^{\gamma_n}\mathbb{E}[\varepsilon_i^2]\mathbb{E}[\varepsilon_j^2]\varepsilon_h\varepsilon_l\varepsilon_m\varepsilon_w.$$

For $i \leq j$, tedious but straightforward calculations included for completeness yield

$$\begin{aligned}
&\mathbb{E}\left[\sum_{h=1}^{i-1}\sum_{l=1}^{i-1}\sum_{m=1}^{j-1}\sum_{w=1}^{j-1} P_{ih}^{\gamma_n}P_{il}^{\gamma_n}P_{jm}^{\gamma_n}P_{jw}^{\gamma_n}\varepsilon_h\varepsilon_l\varepsilon_m\varepsilon_w\right] \\
&= \sum_{l=1}^{i-1}\sum_{h=1}^{i-1}\sum_{m=1}^{j-1}\sum_{w\neq m}^{j-1} P_{il}^{\gamma_n}P_{ih}^{\gamma_n}P_{jm}^{\gamma_n}P_{jw}^{\gamma_n}\mathbb{E}[\varepsilon_l\varepsilon_h\varepsilon_m\varepsilon_w] \\
&\quad + \sum_{l=1}^{i-1}\sum_{h\neq l}^{i-1}\sum_{m\neq l}^{j-1} P_{il}^{\gamma_n}P_{ih}^{\gamma_n}(P_{jm}^{\gamma_n})^2\mathbb{E}[\varepsilon_l]\mathbb{E}[\varepsilon_h\varepsilon_m^2] \\
&\quad + \sum_{l=1}^{i-1}\sum_{h\neq l}^{i-1} P_{il}^{\gamma_n}P_{ih}^{\gamma_n}(P_{jl}^{\gamma_n})^2\mathbb{E}[\varepsilon_l^3]\mathbb{E}[\varepsilon_h] + \sum_{l=1}^{i-1}\sum_{m=1}^{j-1}(P_{il}^{\gamma_n})^2(P_{jm}^{\gamma_n})^2\mathbb{E}[\varepsilon_l^2\varepsilon_m^2] \\
&= \sum_{l=1}^{i-1}\sum_{h=1}^{i-1}\sum_{m=1}^{j-1}\sum_{w\neq m}^{j-1} P_{il}^{\gamma_n}P_{ih}^{\gamma_n}P_{jm}^{\gamma_n}P_{jw}^{\gamma_n}\mathbb{E}[\varepsilon_l\varepsilon_h\varepsilon_m\varepsilon_w] + \sum_{l=1}^{i-1}\sum_{m=1}^{j-1}(P_{il}^{\gamma_n})^2(P_{jm}^{\gamma_n})^2\mathbb{E}[\varepsilon_l^2\varepsilon_m^2]
\end{aligned}$$

$$
\begin{aligned}
&= \sum_{l=1}^{i-1} \sum_{h \neq l}^{i-1} \sum_{m=1}^{j-1} \sum_{w \neq m}^{j-1} P_{il}^{\gamma_n} P_{ih}^{\gamma_n} P_{jm}^{\gamma_n} P_{jw}^{\gamma_n} \mathbb{E}[\varepsilon_l \varepsilon_h \varepsilon_m \varepsilon_w] \\
&\quad + \sum_{l=1}^{i-1} \sum_{m \neq l}^{j-1} \sum_{w \neq m}^{j-1} (P_{il}^{\gamma_n})^2 P_{jm}^{\gamma_n} P_{jw}^{\gamma_n} \mathbb{E}[\varepsilon_l^2 \varepsilon_w] \mathbb{E}[\varepsilon_m] \\
&\quad + \sum_{l=1}^{i-1} \sum_{w \neq l}^{j-1} (P_{il}^{\gamma_n})^2 P_{jl}^{\gamma_n} P_{jw}^{\gamma_n} \mathbb{E}[\varepsilon_l^3] \mathbb{E}[\varepsilon_w] + \sum_{l=1}^{i-1} \sum_{m=1}^{j-1} (P_{il}^{\gamma_n})^2 (P_{jm}^{\gamma_n})^2 \mathbb{E}[\varepsilon_l^2 \varepsilon_m^2] \\
&= \sum_{l=1}^{i-1} \sum_{h \neq l}^{i-1} \sum_{m=1}^{j-1} \sum_{w \neq m}^{j-1} P_{il}^{\gamma_n} P_{ih}^{\gamma_n} P_{jm}^{\gamma_n} P_{jw}^{\gamma_n} \mathbb{E}[\varepsilon_l \varepsilon_h \varepsilon_m \varepsilon_w] + \sum_{l=1}^{i-1} \sum_{m=1}^{j-1} (P_{il}^{\gamma_n})^2 (P_{jm}^{\gamma_n})^2 \mathbb{E}[\varepsilon_l^2 \varepsilon_m^2] \\
&= \sum_{l=1}^{i-1} \sum_{h \neq l}^{i-1} \sum_{m \neq h}^{j-1} \sum_{w \neq m}^{j-1} P_{il}^{\gamma_n} P_{ih}^{\gamma_n} P_{jm}^{\gamma_n} P_{jw}^{\gamma_n} \mathbb{E}[\varepsilon_l \varepsilon_h \varepsilon_m \varepsilon_w] \\
&\quad + \sum_{l=1}^{i-1} \sum_{h \neq l}^{i-1} \sum_{w \notin \{h,l\}}^{j-1} P_{il}^{\gamma_n} P_{ih}^{\gamma_n} P_{jh}^{\gamma_n} P_{jw}^{\gamma_n} \mathbb{E}[\varepsilon_l] \mathbb{E}[\varepsilon_w] \mathbb{E}[\varepsilon_h^2] \\
&\quad + \sum_{l=1}^{i-1} \sum_{h \neq l}^{i-1} P_{il}^{\gamma_n} P_{ih}^{\gamma_n} P_{jh}^{\gamma_n} P_{jl}^{\gamma_n} \mathbb{E}[\varepsilon_l^2] \mathbb{E}[\varepsilon_h^2] + \sum_{l=1}^{i-1} \sum_{m=1}^{j-1} (P_{il}^{\gamma_n})^2 (P_{jm}^{\gamma_n})^2 \mathbb{E}[\varepsilon_l^2 \varepsilon_m^2] \\
&= \sum_{l=1}^{i-1} \sum_{h \neq l}^{i-1} \sum_{m \neq h}^{j-1} \sum_{w \neq m}^{j-1} P_{il}^{\gamma_n} P_{ih}^{\gamma_n} P_{jm}^{\gamma_n} P_{jw}^{\gamma_n} \mathbb{E}[\varepsilon_l \varepsilon_h \varepsilon_m \varepsilon_w] + \sum_{l=1}^{i-1} \sum_{h \neq l}^{i-1} P_{il}^{\gamma_n} P_{ih}^{\gamma_n} P_{jh}^{\gamma_n} P_{jl}^{\gamma_n} \mathbb{E}[\varepsilon_l^2] \mathbb{E}[\varepsilon_h^2] \\
&\quad + \sum_{l=1}^{i-1} \sum_{m=1}^{j-1} (P_{il}^{\gamma_n})^2 (P_{jm}^{\gamma_n})^2 \mathbb{E}[\varepsilon_l^2 \varepsilon_m^2] \\
&= \sum_{l=1}^{i-1} \sum_{h \neq l}^{i-1} \sum_{m \neq h}^{j-1} \sum_{w \notin \{m,h\}}^{j-1} P_{il}^{\gamma_n} P_{ih}^{\gamma_n} P_{jm}^{\gamma_n} P_{jw}^{\gamma_n} \mathbb{E}[\varepsilon_l \varepsilon_h \varepsilon_m \varepsilon_w] \\
&\quad + \sum_{l=1}^{i-1} \sum_{h \neq l}^{i-1} \sum_{m \notin \{h,l\}}^{j-1} P_{il}^{\gamma_n} P_{ih}^{\gamma_n} P_{jm}^{\gamma_n} P_{jh}^{\gamma_n} \mathbb{E}[\varepsilon_l] \mathbb{E}[\varepsilon_m] \mathbb{E}[\varepsilon_h^2] \\
&\quad + 2 \sum_{l=1}^{i-1} \sum_{h \neq l}^{i-1} P_{il}^{\gamma_n} P_{ih}^{\gamma_n} P_{jl}^{\gamma_n} P_{jh}^{\gamma_n} \mathbb{E}[\varepsilon_l^2] \mathbb{E}[\varepsilon_h^2] + \sum_{l=1}^{i-1} \sum_{m=1}^{j-1} (P_{il}^{\gamma_n})^2 (P_{jm}^{\gamma_n})^2 \mathbb{E}[\varepsilon_l^2 \varepsilon_m^2] \\
&= \sum_{l=1}^{i-1} \sum_{h \neq l}^{i-1} \sum_{m \neq h}^{j-1} \sum_{w \notin \{m,h\}}^{j-1} P_{il}^{\gamma_n} P_{ih}^{\gamma_n} P_{jm}^{\gamma_n} P_{jw}^{\gamma_n} \mathbb{E}[\varepsilon_l \varepsilon_h \varepsilon_m \varepsilon_w] \\
&\quad + 2 \sum_{l=1}^{i-1} \sum_{h \neq l}^{i-1} P_{il}^{\gamma_n} P_{ih}^{\gamma_n} P_{jl}^{\gamma_n} P_{jh}^{\gamma_n} \mathbb{E}[\varepsilon_l^2] \mathbb{E}[\varepsilon_h^2] + \sum_{l=1}^{i-1} \sum_{m=1}^{j-1} (P_{il}^{\gamma_n})^2 (P_{jm}^{\gamma_n})^2 \mathbb{E}[\varepsilon_l^2 \varepsilon_m^2] \\
&= \sum_{l=1}^{i-1} \sum_{h \neq l}^{i-1} \sum_{m \neq h}^{j-1} \sum_{w \notin \{m,h,l\}}^{j-1} P_{il}^{\gamma_n} P_{ih}^{\gamma_n} P_{jm}^{\gamma_n} P_{jw}^{\gamma_n} \mathbb{E}[\varepsilon_l \varepsilon_h \varepsilon_m] \mathbb{E}[\varepsilon_w]
\end{aligned}
$$

$$
\begin{aligned}
&+\sum_{l=1}^{i-1}\sum_{h\neq l}^{i-1}\sum_{m\notin\{h,l\}}^{j-1} P_{il}^{\gamma_n}P_{ih}^{\gamma_n}P_{jm}^{\gamma_n}P_{jl}^{\gamma_n}\mathbb{E}[\varepsilon_l^2]\mathbb{E}[\varepsilon_h]\mathbb{E}[\varepsilon_m]\\
&+\sum_{l=1}^{i-1}\sum_{h\neq l}^{i-1} P_{il}^{\gamma_n}P_{ih}^{\gamma_n}(P_{jl}^{\gamma_n})^2\mathbb{E}[\varepsilon_l^3]\mathbb{E}[\varepsilon_h] + 2\sum_{l=1}^{i-1}\sum_{h\neq l}^{i-1} P_{il}^{\gamma_n}P_{ih}^{\gamma_n}P_{jl}^{\gamma_n}P_{jh}^{\gamma_n}\mathbb{E}[\varepsilon_l^2]\mathbb{E}[\varepsilon_h^2]\\
&+\sum_{l=1}^{i-1}\sum_{m=1}^{j-1}(P_{il}^{\gamma_n})^2(P_{jm}^{\gamma_n})^2\mathbb{E}[\varepsilon_l^2\varepsilon_m^2]\\
=&2\sum_{l=1}^{i-1}\sum_{h\neq l}^{i-1} P_{il}^{\gamma_n}P_{ih}^{\gamma_n}P_{jl}^{\gamma_n}P_{jh}^{\gamma_n}\mathbb{E}[\varepsilon_l^2]\mathbb{E}[\varepsilon_h^2] + \sum_{l=1}^{i-1}\sum_{m=1}^{j-1}(P_{il}^{\gamma_n})^2(P_{jm}^{\gamma_n})^2\mathbb{E}[\varepsilon_l^2\varepsilon_m^2]\\
=&2\sum_{l=1}^{i-1}\sum_{h\neq l}^{i-1} P_{il}^{\gamma_n}P_{ih}^{\gamma_n}P_{jl}^{\gamma_n}P_{jh}^{\gamma_n}\mathbb{E}[\varepsilon_l^2]\mathbb{E}[\varepsilon_h^2] + \sum_{l=1}^{i-1}\sum_{m\neq l}^{j-1}(P_{il}^{\gamma_n})^2(P_{jm}^{\gamma_n})^2\mathbb{E}[\varepsilon_l^2]\mathbb{E}[\varepsilon_m^2]\\
&+\sum_{l=1}^{i-1}(P_{il}^{\gamma_n})^2(P_{jl}^{\gamma_n})^2\mathbb{E}[\varepsilon_l^4]\\
=&2\sum_{h=1}^{i-1}\sum_{l\neq h} P_{ih}^{\gamma_n}P_{il}^{\gamma_n}P_{jh}^{\gamma_n}P_{jl}^{\gamma_n}\mathbb{E}[\varepsilon_h^2]\mathbb{E}[\varepsilon_l^2]\\
&+\sum_{h=1}^{i-1}(P_{ih}^{\gamma_n})^2(P_{jh}^{\gamma_n})^2\mathrm{Var}[\varepsilon_h^2] + \sum_{h=1}^{i-1}\sum_{l=1}^{j-1}(P_{ih}^{\gamma_n})^2(P_{jl}^{\gamma_n})^2\mathbb{E}[\varepsilon_h^2]\mathbb{E}[\varepsilon_l^2],
\end{aligned}
$$

where $\mathrm{Var}[\varepsilon_h^2] \coloneqq \mathbb{E}[\varepsilon_h^4] - \mathbb{E}[\varepsilon_h^2]\mathbb{E}[\varepsilon_h^2]$.

Defining $q \coloneqq \min\{i,j\}$, the above display in turn implies

$$
\begin{aligned}
\mathbb{E}[\mathcal{V}_n^4] =&32\sum_{i=2}^{n}\mathbb{E}[\varepsilon_i^2]\sum_{j=2}^{n}\mathbb{E}[\varepsilon_j^2]\sum_{h=1}^{q-1}\sum_{l\neq h} P_{ih}^{\gamma_n}P_{il}^{\gamma_n}P_{jh}^{\gamma_n}P_{jl}^{\gamma_n}\mathbb{E}[\varepsilon_h^2]\mathbb{E}[\varepsilon_l^2]\\
&+16\sum_{i=2}^{n}\mathbb{E}[\varepsilon_i^2]\sum_{j=2}^{n}\mathbb{E}[\varepsilon_j^2]\sum_{h=1}^{q-1}(P_{ih}^{\gamma_n})^2(P_{jh}^{\gamma_n})^2\mathrm{Var}[\varepsilon_h^2]\\
&+16\sum_{i=2}^{n}\mathbb{E}[\varepsilon_i^2]\sum_{j=2}^{n}\mathbb{E}[\varepsilon_j^2]\sum_{h=1}^{i-1}\sum_{l=1}^{j-1}(P_{ih}^{\gamma_n})^2(P_{jl}^{\gamma_n})^2\mathbb{E}[\varepsilon_h^2]\mathbb{E}[\varepsilon_l^2]\\
\equiv&\mathfrak{A}_n + \mathfrak{B}_n + s_n^4.
\end{aligned}
$$

In order to establish the convergence in Equation (A.24), it is sufficient to show that

$\frac{\mathfrak{A}_n+\mathfrak{B}_n}{s_n^4} \to 0$. To this end, note that

$$\begin{aligned}
\mathfrak{A}_n &\leq C\sum_{i=1}^{n}\sum_{j=1}^{n}\sum_{h=1}^{q-1}\sum_{l\neq h} P_{ih}^{\gamma_n}P_{il}^{\gamma_n}P_{jh}^{\gamma_n}P_{jl}^{\gamma_n} \\
&= C\left(\sum_{i=1}^{n}\sum_{j\neq i}\sum_{h=1}^{q-1}\sum_{l\neq h} P_{ih}^{\gamma_n}P_{il}^{\gamma_n}P_{jh}^{\gamma_n}P_{jl}^{\gamma_n} + \sum_{i=1}^{n}\sum_{h=1}^{q-1}\sum_{l\neq h}(P_{ih}^{\gamma_n})^2(P_{il}^{\gamma_n})^2\right) \\
&\leq C\left(\sum_{i=1}^{n}\sum_{j<i}\sum_{h<j}\sum_{l\neq h} P_{ih}^{\gamma_n}P_{il}^{\gamma_n}P_{jh}^{\gamma_n}P_{jl}^{\gamma_n} + r_n\right) \\
&\leq C\left(\sum_{i=1}^{n}\sum_{j<i}\sum_{h<j}\sum_{l<h} P_{ih}^{\gamma_n}P_{il}^{\gamma_n}P_{jh}^{\gamma_n}P_{jl}^{\gamma_n} + r_n\right) \\
&\leq C\left(\left|\sum_{i=1}^{n}\sum_{j<i}\sum_{h<j}\sum_{l<h} P_{ih}^{\gamma_n}P_{il}^{\gamma_n}P_{jh}^{\gamma_n}P_{jl}^{\gamma_n}\right| + r_n\right) \\
&\leq Cr_n
\end{aligned} \tag{A.25}$$

The first inequality follows from Assumption 1. The second inequality follows from Lemma 1 (vi). The third inequality follows from the symmetry of P^{γ_n}. The fifth inequality follows from Lemma 2. Next,

$$\mathfrak{B}_n \leq C\sum_{i=1}^{n}\sum_{j=1}^{n}\sum_{h=1}^{q-1}(P_{ih}^{\gamma_n})^2(P_{jh}^{\gamma_n})^2 \leq Cr_n, \tag{A.26}$$

the first inequality following from Assumption 1 and the second from Lemma 1 (vi). Thus,

$$\frac{\mathfrak{A}_n+\mathfrak{B}_n}{s_n^4} \leq C\frac{r_n}{\left(\sum_{i=1}^{n}\sum_{j\neq i}(P_{ij}^{\gamma_n})^2\right)^2} \leq Cr_n^{1-2} \to 0. \tag{A.27}$$

where the second inequality follows from Equation (A.1) and the convergence from Assumption 2 and Assumption 3. Equation (A.27) verifies the condition in Equation (A.24), which in turn implies that the condition condition in Equation (A.21) holds.

Having verified the conditions in Equation (A.20) and Equation (A.21) we conclude that $RJAR^*_{\gamma_n} \xrightarrow{d} \mathcal{N}[0,1]$. By Lemma 4 $|\hat{\Phi}_{\gamma_n} - \Phi_{\gamma_n}| \xrightarrow{p} 0$ and hence also $\hat{\Phi}_{\gamma_n}/\Phi_{\gamma_n} \xrightarrow{p} 1$ under Assumptions 1 and 3. Hence, under the null hypothesis, the continuous mapping theorem implies that $RJAR_{\gamma_n}(\beta_0) \xrightarrow{d} \mathcal{N}[0,1]$.

□

B Simulation results with heteroskedastic errors

We extend the simulations in the main text to allow for heteroskedasticity in the error terms. As in the main text, the DGP is given by

$$y_i = X_i\beta + \varepsilon_i \tag{B.1a}$$

$$X_i = Z_i'\pi + v_i, \tag{B.1b}$$

for $i = 1, \ldots, n = 100$. The IVs Z_i are independent and identically Gaussian with mean 0 and $\mathrm{Var}[Z_{il}] = 0.3$ and $\mathrm{Corr}[Z_{il}, Z_{im}] = 0.5^{|l-m|}$. As in Guggenberger et al. (2023), the error terms are given by

$$\varepsilon_i = (\sigma_\varepsilon + ||Q_\varepsilon \tilde{Z}_i||_2)\eta_{1i}$$

$$v_i = (\sigma_v + ||Q_v \tilde{Z}_i||_2)\eta_{2i},$$

where $\tilde{Z}_i$ is the 4×1 vector consisting of the first 4 elements of Z_i, $\sigma_\varepsilon = \sqrt{2}$, $\sigma_v = 1$, Q_ε and Q_v are 4×4 matrices controlling the degree of heteroskedasticity in the first and second stage, respectively,[4] and

$$[\eta_{1i}, \eta_{2i}]' \sim \mathcal{N}\left[\left[\begin{smallmatrix}0\\0\end{smallmatrix}\right], \left[\begin{smallmatrix}1 & 0.6\\0.6 & 1\end{smallmatrix}\right]\right].$$

The $[\eta_{1i}, \eta_{2i}]$ are mutually independent as well as independent of the $\tilde{Z}_i$. We set

$$Q_\varepsilon = \left[\begin{smallmatrix}2 & 0.8 & 0.6 & 0.4\\ 0.3 & 1.5 & 0.9 & 0.3\\ 0.8 & 0.6 & 1.9 & 0.2\\ 0.4 & 0.3 & 0.2 & 1.1\end{smallmatrix}\right] \quad \text{and} \quad Q_v = I_4, \tag{B.2}$$

following Guggenberger et al. (2023).[5]

$\pi = \zeta\kappa$, where κ is a vector of zeros and ones that varies with the type of DGP considered (sparse or dense, as modelled below), and ζ is some scalar that ensures that for a given value of μ^2, the following relationship is satisfied:

$$\mu^2 = \frac{n\pi'\mathbb{E}[Z_i Z_i']\pi}{\sigma_v^2}.$$

[4]The simulation setup in the main text corresponds to the case where Q_ε and Q_v are set equal to the 4×4 matrix of zeros.

[5]Our matrix Q_ε corresponds to Q_ε in Guggenberger et al. (2023, Equation (4.9)) with ϱ (in their notation) set equal to 0.1.

We note that in this heteroskedastic context, μ^2 is only used to ensure that the coefficients on the IVs are the same as in the homoskedastic DGP considered in Section 4 in the main text, and does not measure identification strength.

As in the main text, we consider both a sparse first stage and a dense first stage. Sparsity in the first stage is modelled by setting $\kappa = [\iota_5', 0_{k_n-5}']'$, where ι_q is a $q \times 1$ vector of ones, and 0_q is a $q \times 1$ vector of zeros. Density in the first stage is modelled by setting $\kappa = [\iota_{0.4k_n}', 0_{0.6k_n}']'$. We consider $k_n = 30, 90, 190$. In the context of Assumption 3, we search over values greater than 1 when choosing γ_n^* in case $r_n < k_n$. The variance estimator of MS occasionally yields a negative value. These cases are conservatively interpreted as a failure to reject the null hypothesis. As recommended by BCCH, $c_{BCCH} = 1.1$. As in the simulation section in CT, we set $\theta = 0.05$. The number of Monte Carlo replications is 10,000.

B.1 Size

Figure 1 shows the simulation results with a sparse first stage for tests of size 0.01 to 0.99, that is the rejection frequency under $H_0 : \beta_0 = 1$. The results are similar to the ones of the homoskedastic DGP considered in Section 4 in the main text, although the AR test of CT exhibts some mild size distortion.

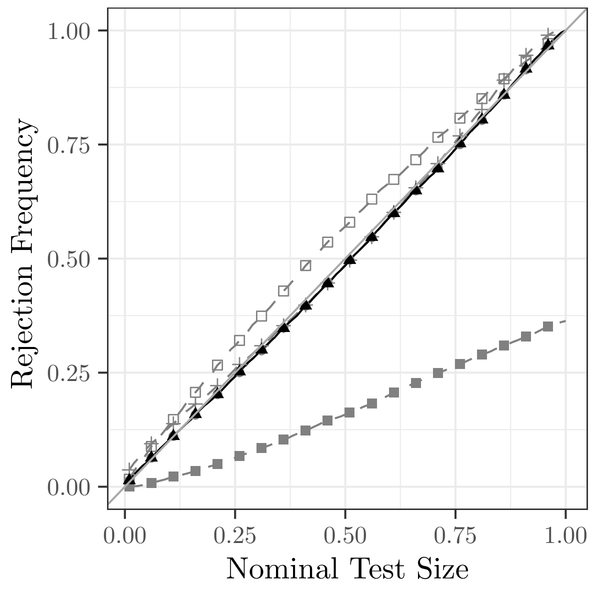

(a) $k_n = 30$

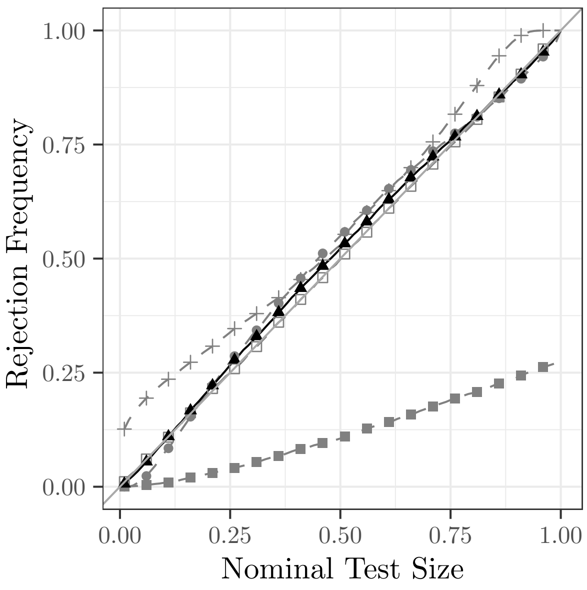

(b) $k_n = 90$

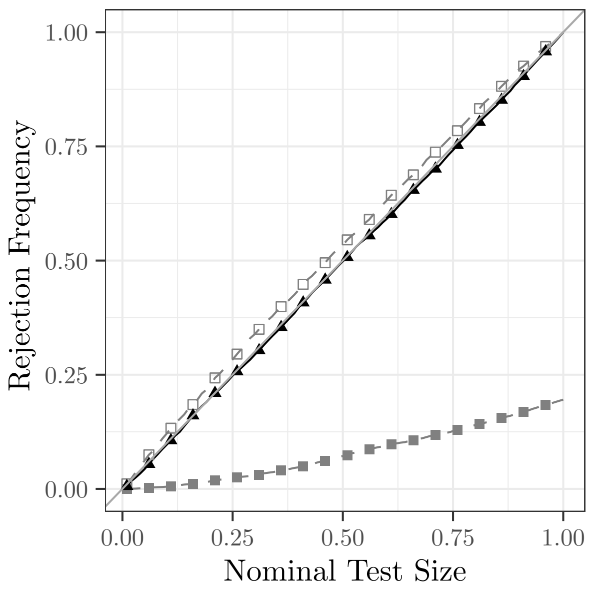

(c) $k_n = 190$

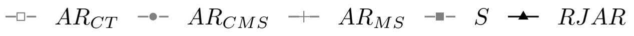

Figure 1: PP Plots for Sparse IVs, heteroskedastic errors, $\beta = 1$, $\mu^2 = 0$, $H_0 : \beta_0 = 1$.

B.2 Power

Figures 2–4 show the power of the tests when the number of IVs and the sparsity pattern of the first stage is varied. It is still the case that $H_0 : \beta_0 = 1$. We report the results for $\mu^2 = 180$, as the power for $\mu^2 = 30$ is very low for all tests. The results confirm that also in this heteroskedastic setup, the RJAR test is as powerful as existing methods whenever these are applicable, and sometimes much more powerful.

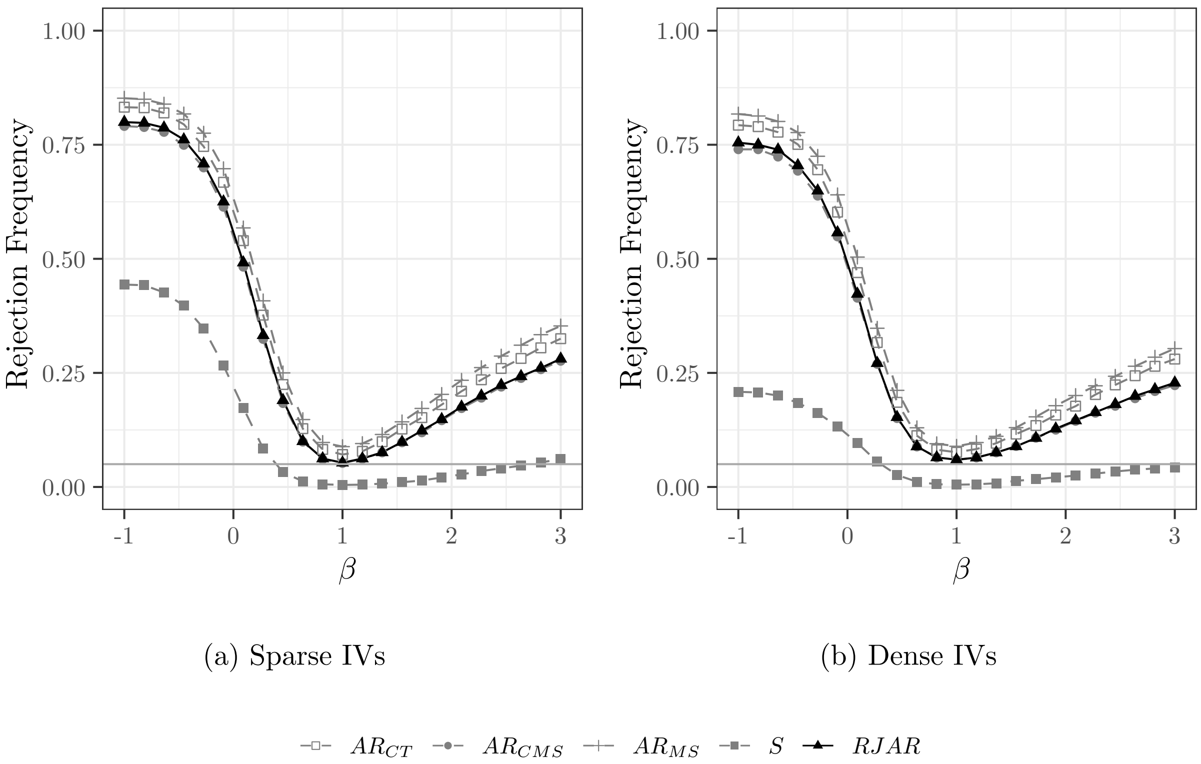

Figure 2: Power curves for 30 IVs and heteroskedastic errors. Nominal test size of 5% indicated by the grey horizontal line. $\mu^2 = 180$, $H_0 : \beta_0 = 1$.

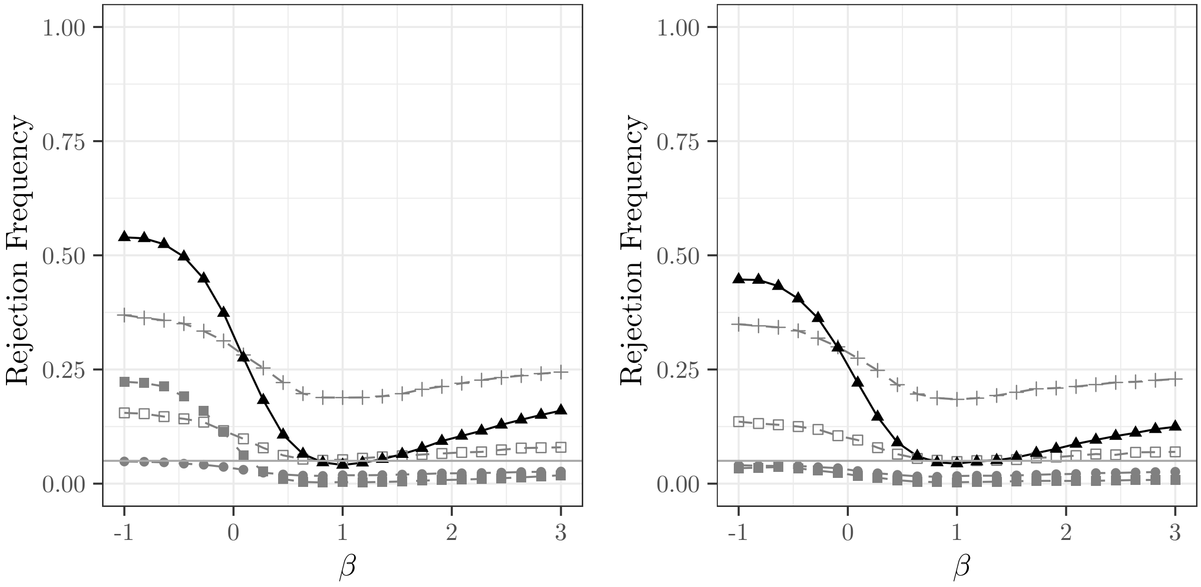

(a) Sparse IVs

(b) Dense IVs

Figure 3: Power curves for 90 IVs and heteroskedastic error terms. Nominal test size of 5% indicated by the grey horizontal line. $\mu^2 = 180$, $H_0 : \beta_0 = 1$.

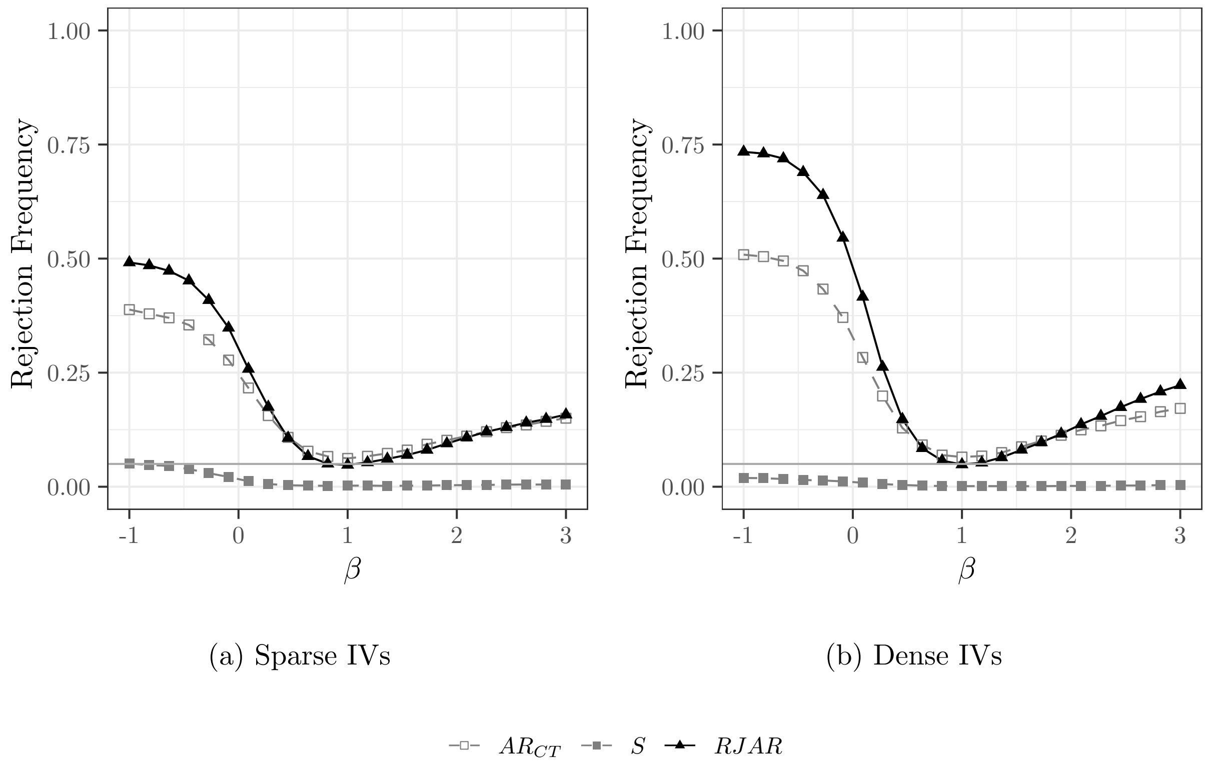

Figure 4: Power curves for 190 IVs and heteroskedastic error terms. Nominal test size of 5% indicated by the grey horizontal line. $\mu^2 = 180$, $H_0 : \beta_0 = 1$.

C Simulation results for projection inference

To study projection inference, we use the simulation setup for two endogenous variables of Guggenberger et al. (2023).

The DGP is given by

$$y_i = \beta_1 X_{i1} + \beta_2 X_{i2} + \varepsilon_i$$

$$X_{i1} = Z_i' \pi_1 + v_{i1}$$

$$X_{i2} = Z_i' \pi_2 + v_{i2},$$

for $i = 1, \ldots, n = 250$. Inference is conducted on β_1 by projecting out X_{i2}. $\beta_2 = 0$, $\pi_1 = \tilde{\iota}_{k_n} \zeta_1 / (4n)^{1/2}$, $\pi_2 = \iota_{k_n} \zeta_2 / (4n)^{1/2}$, $\tilde{\iota}_{k_n} = [\iota'_{k_n/2}, -\iota'_{k_n/2}]'$, $\zeta_1, \zeta_2 \in \{4, 40\}$ (weak and

strong IVs), and $Z_i \overset{i.i.d.}{\sim} \mathcal{N}[0, I_{k_n}]$. The error terms are given by

$$\varepsilon_i = (||Q_\varepsilon \tilde{Z}_i||_F)\eta_{1i}$$

$$v_{1i} = (||Q_v \tilde{Z}_i||_F)\eta_{2i}$$

$$v_{2i} = (||Q_v \tilde{Z}_i||_F)\eta_{3i},$$

where $\tilde{Z}_i$ is the 4×1 vector consisting of the first 4 elements of Z_i, the 4×4 matrices Q_ε, Q_v control the type and degree of heteroskedasticity and are set as in (B.2), and

$$[\eta_{1i}, \eta_{2i}, \eta_{3i}]' \sim \mathcal{N}\left[\begin{bmatrix} 0 \\ 0 \\ 0 \end{bmatrix}, \begin{bmatrix} 1 & 0.8 & 0.8 \\ 0.8 & 1 & 0.3 \\ 0.8 & 0.3 & 1 \end{bmatrix}\right].$$

We consider $k_n = 30$ (the number of IVs for which all approaches have correct size in the simulations reported in the main text) for three combinations of π_1 and π_2 (strong, strong), (weak, strong), (weak, weak). The variance estimator of MS occasionally yields a negative value. These cases are conservatively interpreted as a failure to reject the null hypothesis. c_{BCCH}, and θ are set as in the main text. The number of Monte Carlo replications is 10,000.

Figure 5 shows the power of the projection tests for $H_0 : \beta_{1,0} = 0$. The tests have similar power except for the Sup Score test of BCCH, which has very low power. This is likely due to the dense parameterisation of the first-stage projection vectors in the DGP of Guggenberger et al. (2023). The AR test of CT exhibits some size distortions. This is likely due to the heteroskedasticity in the error terms.

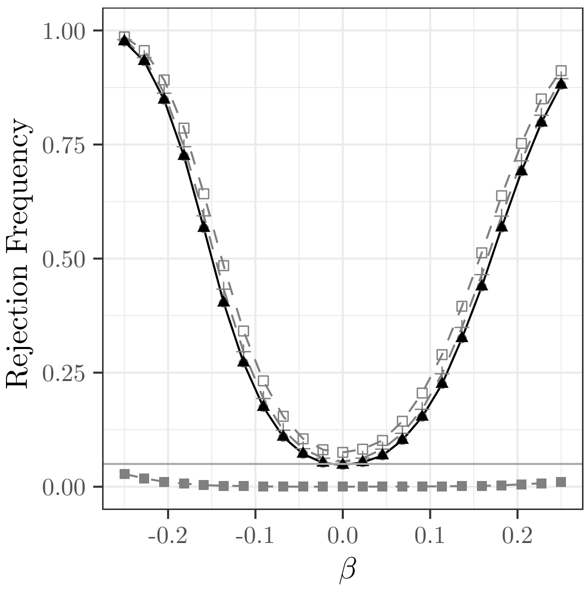

(a) Strong ($\xi_1 = 40$, $\xi_2 = 40$)

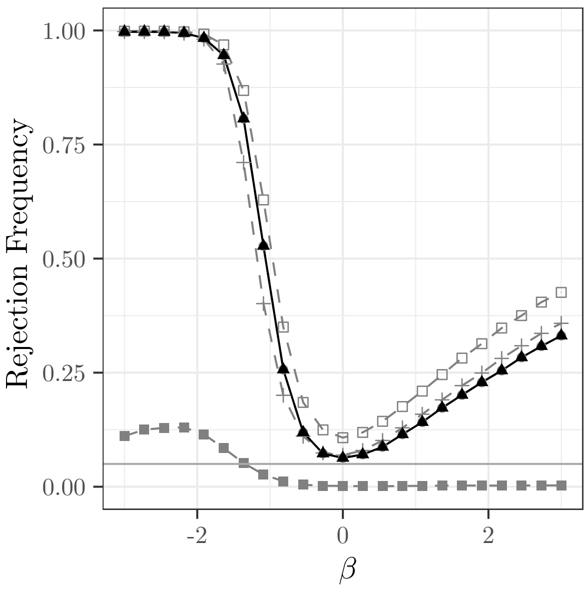

(b) Mixed strength ($\xi_1 = 4$, $\xi_2 = 40$)

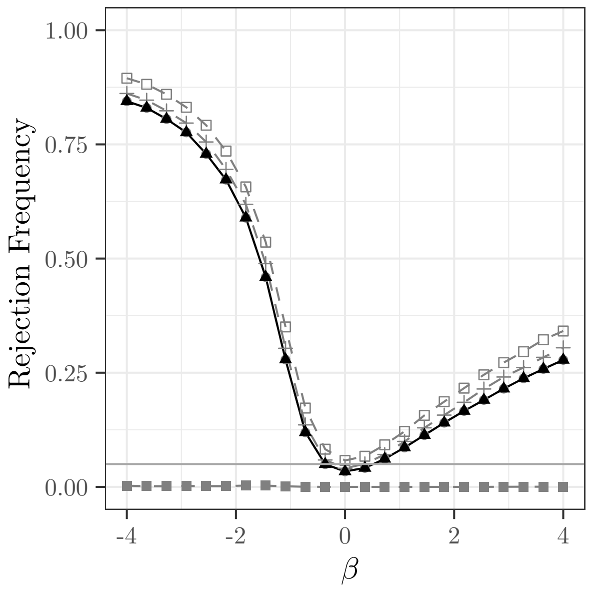

(c) Weak ($\xi_1 = 4$, $\xi_2 = 4$)

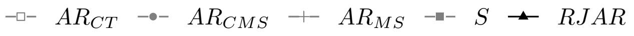

Figure 5: Power curves for projection inference with heteroskedastic error terms. Nominal test size of 5% indicated by the grey horizontal line. $H_0 : \beta_{1,0} = 0$.

D Simulation results with uncorrelated IVs

The same DGP as in the main text is used, except that $Z_i \overset{i.i.d.}{\sim} \mathcal{N}[0, I_{k_n}]$.

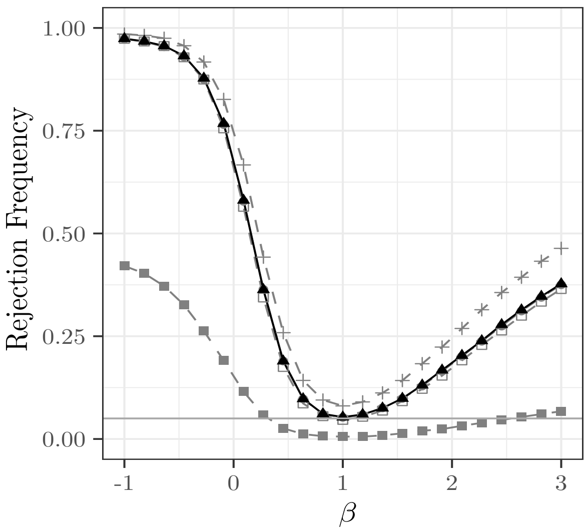

(a) $\mu^2 = 30$, sparse IVs

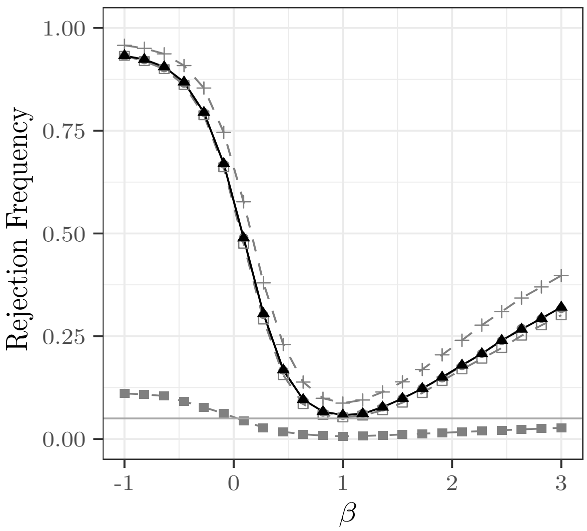

(b) $\mu^2 = 30$, dense IVs

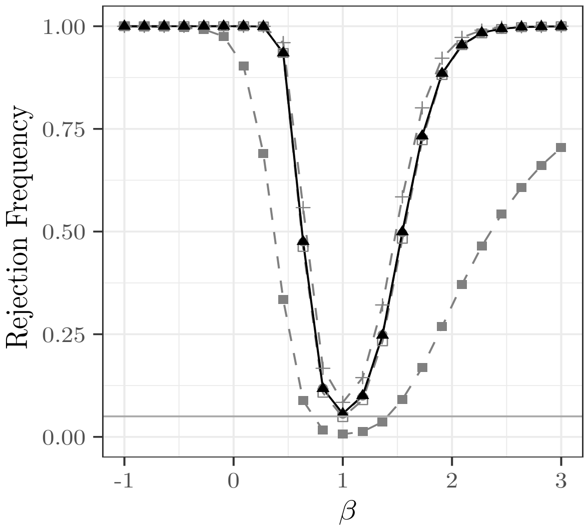

(c) $\mu^2 = 180$, sparse IVs

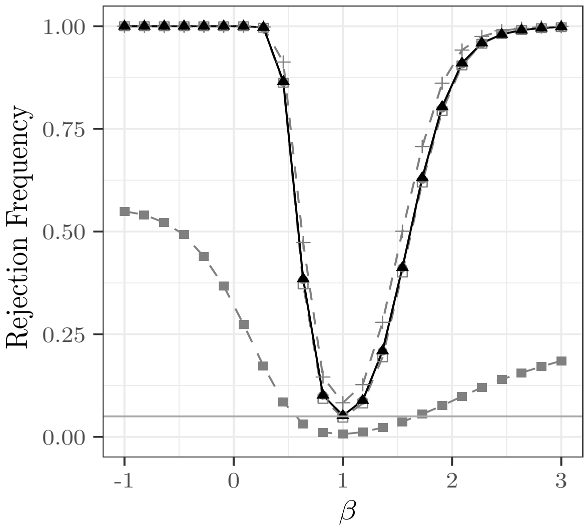

(d) $\mu^2 = 180$, dense IVs

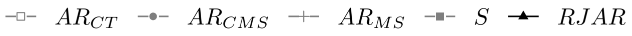

Figure 6: Power curves for 30 independent IVs. Nominal test size of 5% indicated by the grey horizontal line. $H_0 : \beta_0 = 1$.

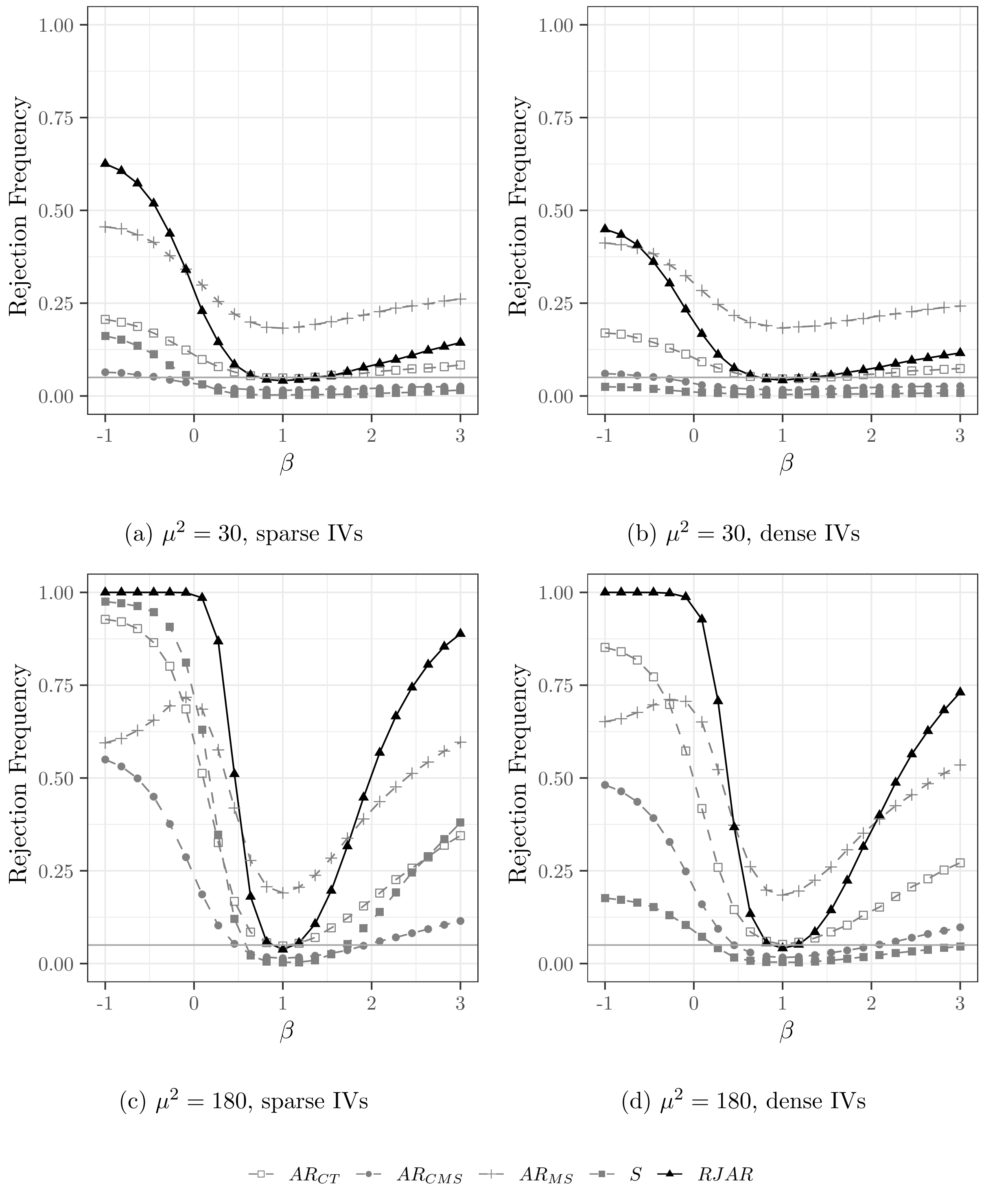

Figure 7: Power curves for 90 independent IVs. Nominal test size of 5% indicated by the grey horizontal line. $H_0 : \beta_0 = 1$.

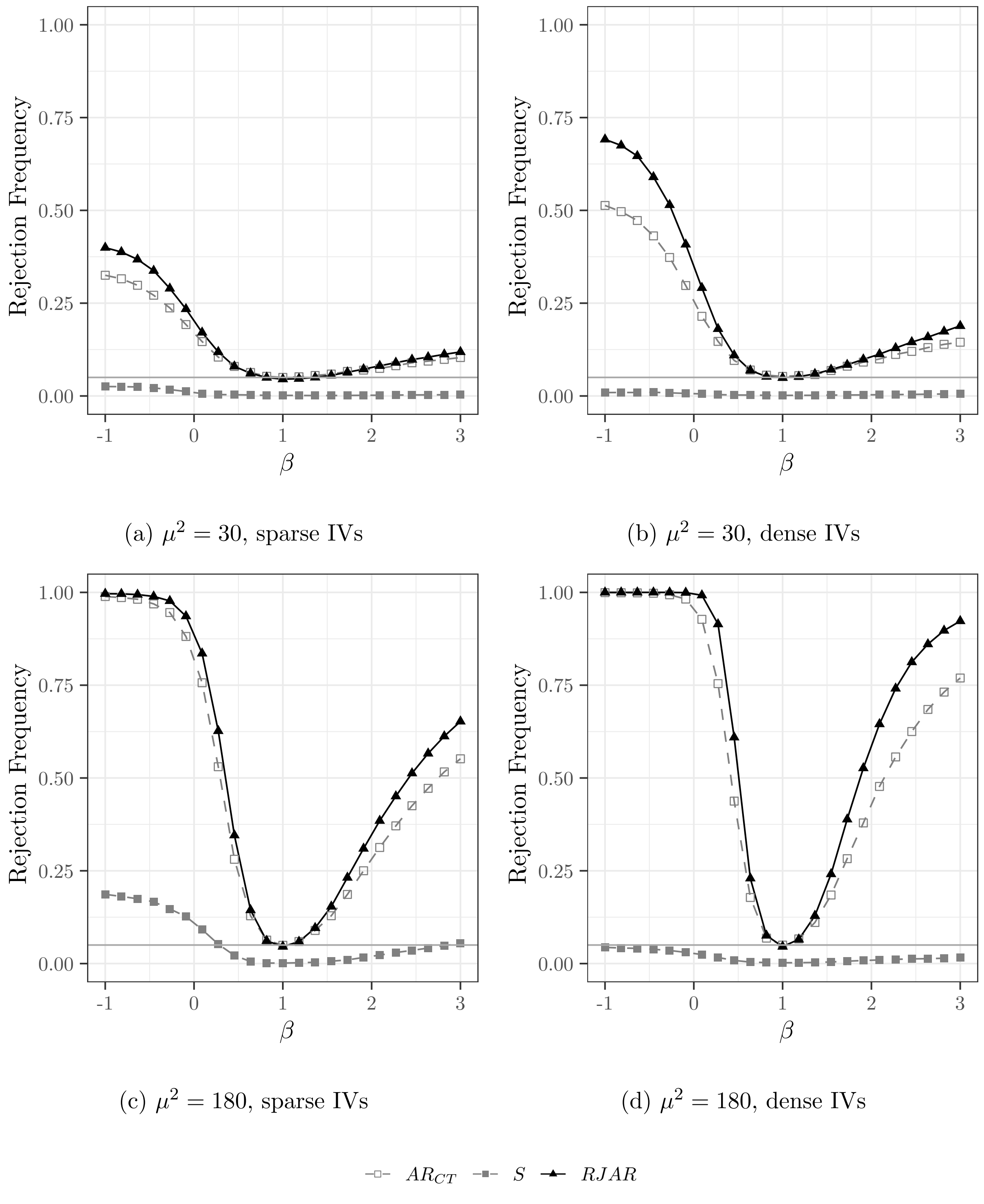

Figure 8: Power curves for 190 independent IVs. Nominal test size of 5% indicated by the grey horizontal line. $H_0 : \beta_0 = 1$.

E Size of AR test of MS with $n = 1000$ and $k_n = 900$

The same DGP as in the main text is used, except that $k_n = 900$ and $n = 1,000$.

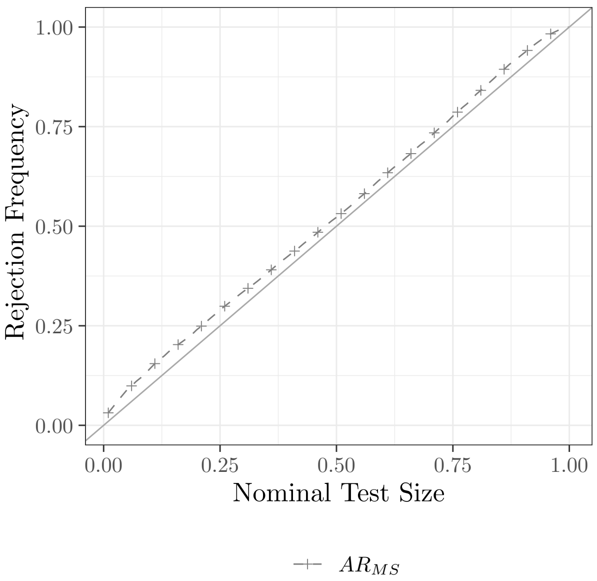

Figure 9: PP Plots for AR test of MS with sparse IVs, homoskedastic errors, $\beta = 1$, $\mu^2 = 0$, $H_0 : \beta_0 = 1$, $n = 1000$, $k_n = 900$.

References

Bai, Z. and J. Silverstein (2010). *Spectral analysis of large dimensional random matrices*, Volume 20. Springer.

Chao, J., J. Hausman, W. Newey, N. Swanson, and T. Woutersen (2014). Testing overidentifying restrictions with many instruments and heteroskedasticity. *Journal of Econometrics 178*, 15–21.

Chao, J., N. Swanson, J. Hausmann, W. Newey, and T. Woutersen (2012). Asymptotic Distribution of JIVE in a Heteroskedastic IV Regression with Many Instruments. *Econometric Theory 28*, 42–86.

Guggenberger, P., F. Kleibergen, and S. Mavroeidis (2023). A powerful anderson-rubin test in linear instrumental variables regression with conditional heteroskedasticity. *Econometric Theory (Forthcoming)*, 1–59.

Hall, P. and C. Heyde (1980). *Martingale Limit Theory and Its Application.* Academic Press.

Hansen, C. and D. Kozbur (2014). Instrumental variables estimation with many weak instruments using regularized JIVE. *Journal of Econometrics 182*(2), 290–308.

Lütkepohl, H. (1996). *Handbook of Matrices.* Wiley.
